# Composite Materials Based on a Zr^4+^ MOF and Aluminosilicates for the Simultaneous Removal of Cationic and Anionic Dyes from Aqueous Media

**DOI:** 10.3390/molecules28020815

**Published:** 2023-01-13

**Authors:** Petros Georgianos, Anastasia D. Pournara, Evangelos K. Andreou, Gerasimos S. Armatas, Manolis J. Manos

**Affiliations:** 1 Department of Chemistry, University of Ioannina, GR-45110 Ioannina, Greece; 2 Department of Materials Science and Technology, University of Crete, GR-70013 Heraklion, Greece; 3 Institute of Materials Science and Computing, University Research Center of Ioannina, GR-45110 Ioannina, Greece

**Keywords:** metal–organic frameworks, composite materials, dyes’ sorption, methylene blue, methyl orange, zeolite, clay, sorption column, alginate beads

## Abstract

Environmental pollution has been a reality for many decades, with its contamination intensifying daily due to rapid urbanization and the ever-increasing world population. Dyes, and especially synthetic ones, constitute a category of pollutants that not only affect the quality of water but also exhibit high toxicity toward living organisms. This study was thoroughly planned to explore the removal of two toxic dyes, namely the methylene blue (MB) and methyl orange (MO) compounds from contaminated aqueous media. For this purpose, we designed and synthesized two new composite materials based on ammonium-functionalized Zr^4+^ MOF (MOR-1 or UiO-66-NH_3_^+^) and naturally occurring sorbents, such as bentonite and clinoptilolite. The composite materials displayed exceptional sorption capability toward both MB^+^ and MO^−^ ions. A key finding of this study was the high efficiency of the composite materials to simultaneously remove MB^+^ and MO^−^ under continuous flow conditions, also showing regeneration capability and reusability, thus providing an alternative to well-known mixed bed resins.

## 1. Introduction

In recent centuries, the industrialization of human societies has led to massive urbanization in most regions on planet Earth. Consequently, different kinds of industries have developed, which have served people’s needs in various aspects of their daily life. However, the proliferation of people’s needs for products and the rapid increase of industrial production have resulted in the generation of hazardous waste and toxic gases, alongside the production of the desired products. Aqueous wastes, originating from industry or from human processes, when released into the environment without any kind of treatment, cause serious water pollution. 

Over the years, various pollutants, such as pharmaceuticals and personal care products (PCPs), pesticides, dyes, oil, and aromatic and/or organic compounds, have been detected in aquatic environments [1]. Interestingly, dyes and pigments, which are widely used in textile industries, tanneries, paper mills, and plastics production, are the most abundant pollutants in aquatic environments. It is estimated that approximately 100,000 commercially available dyes are produced each year at a rate of 8 × 10^5^ tons per year, with 15% of the dyes produced typically being released as wastewater to water bodies [2,3,4]. Water quality is greatly affected by color, and even a small percentage of dye can be visible and cause a color sensation in the water [5]. These seemingly ‘innocent pollutants’ are harmful to fish and other aquatic organisms, are potentially carcinogenic, and can cause acute or chronic diseases to exposed living beings [6,7]. Moreover, dyes exhibit resistance to light, heat, and oxidizing agents, a fact that is derived from their complex aromatic molecular structures. On that account, the protection of the earth’s water resources becomes vital and has been facilitated through the development of effective and efficient separation and purification techniques. 

So far, the reported techniques for the treatment of dye-contaminated aquatic sources include sorption, flocculation, electrolysis, photocatalysis, and biodegradation [8,9,10,11]. Among these methods, sorption is the simplest and most efficient one adopted for these purposes [12]. Thus, in the last decades, special attention has been paid to the research on nanoporous materials, as it has been found that they are good sorbents and play an important role in separation and purification processes. The relatively new class of highly crystalline porous solids, metal–organic frameworks (MOFs), is constantly gaining ground in various research fields, and especially in applications regarding sorption removal and the separation/purification of contaminated wastewater [13,14,15,16].

In our previous work, we reported the successful removal of the acidic/anionic dye methyl orange (MO^−^) with the [Zr_6_O_4_(OH)_4_(NH_3_^+^-BDC)_6_]Cl_6_·solvent (ΜOR-1 or UiO-66-NH_3_^+^), where NH_2_-BDC^2−^ = 2-amino-terephthalate, in its composite form with alginic acid (HA) [12]. Although MOR-1-HA has been shown to be an excellent sorbent toward MO^−^, the results were rather discouraging when the cationic dye methylene blue (MB^+^) was used as the target pollutant, confirming our prediction that the sorption mechanism involves an anion-exchange process [12]. To this end, we decided to improve the sorbent functionality to enable the removing of both anionic and cationic species. Given the fact that MOR-1-HA could easily exchange its Cl^−^ with MO^−^, the goal was to design a composite which consisted of MOR-1 and a secondary unit that could capture cationic species. Clays and zeolites were two promising classes of materials since they have been extensively applied in the removal of cationic dyes from wastewater, indicating superior sorption behavior with great cation-exchange properties [17,18]. Herein, we present two composite materials based on the anion-exchanging material ΜOR-1, combined with natural inorganic cation-exchange materials, specifically bentonite (clay) and clinoptilolite (zeolite), together with alginic acid—a natural organic polymer. The composite materials **(MOR-1/Bentonite)-HA** and **(MOR-1/Clinoptilolite)-HA** exhibited an exceptional capability to remove MO^−^ and MB^+^ dyes from water solutions under various conditions. Additionally, we synthesized two composite sorbents based on the above-mentioned materials, calcium alginate and Fe_3_O_4_, which in the form of beads could be successfully employed in ion-exchange columns. It is worth mentioning that such columns combine the capability for the simultaneous reduction of the concentrations of mixed MO^−^/MB^+^ dyes in aqueous solutions and regeneration capacity reusability, thus offering an alternative to the use of mixed bed resins [19].

## 2. Results

### 2.1. Synthesis of the Composite Materials

**Figure 1:**
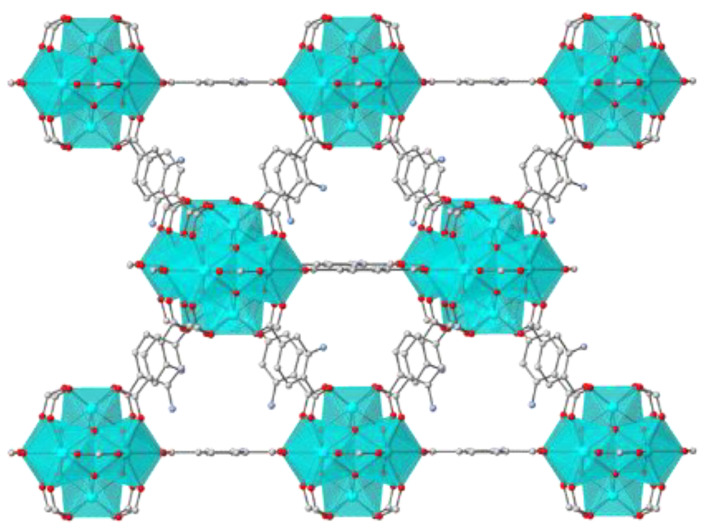
Representation of the structure of MOR-1. Color code: C, grey; O, red; N, blue; Zr, cyan.

In our group, several ammonium-functionalized Zr^4+^ MOFs have been reported and widely used as sorbents for the removal of various anionic species [12,20,21,22,23,24,25]. However, these MOFs could not be applied in solutions containing cationic pollutants without the previous deprotonation of the amine group [26], which means that the material could not further uptake anions. The principal idea in this study was to design composite multifunctional sorbents based on MOR-1 (Figure 1), an excellent anion sorbent, and widely available aluminosilicate materials, such as bentonite and clinoptilolite (Figure S1) that are well-known for their highly efficient cation-exchange properties [27,28,29,30]. Thus, the resulted materials were capable of simultaneously removing both anionic and cationic pollutants.

**Figure 2:**
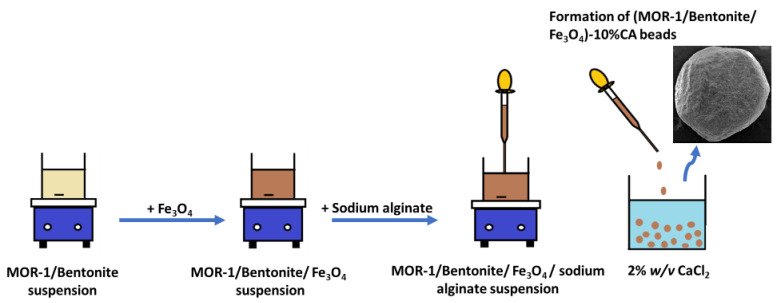
Schematic representation of the preparation of **(MOR-1/Bentonite/Fe_3_O_4_)-10%CA beads**.

The synthesis of these composite materials was carried out in aqueous solutions of sodium alginate where MOR-1 and bentonite or clinoptilolite were mixed in a weight percentage ratio of 1:1. Within these solutions, layers of alginate anions covered the particles of the materials and the addition of concentrated acetic acid led to the precipitation of the **(MOR-1/Bentonite)-HA** and **(MOR-1/Clinoptilolite)-HA (HA = alginic acid)** composites. The weight percentage of alginate’s contribution in each composite was only 2% and did not affect the sorption process. These materials were proven excellent sorbents via batch sorption studies (see below). However, they were isolated in the form of fine powder, and thus they were not suitable to be used as stationary phases in columns. To this end, we prepared composite materials, namely **(MOR-1/Bentonite/Fe_3_O_4_)-10%CA** and **(MOR-1/Clinoptilolite/Fe_3_O_4_)-10%CA (CA = calcium alginate)** in the form of beads (Figure 2), which contained more alginate content (10%) as well as Fe_3_O_4_ particles. The latter component not only allowed an easy recovery of the beads by applying an external magnetic field (Figure S2), but also seemed to enhance the mechanical strength of the beads, facilitating the unhindered flow of water solutions through the stationary phase of the column. We should note that the significantly increased alginate content in the beads, compared to that of the powder form of the composites, may result in slower sorption kinetics; however, the isolation of composites in the form of beads requires a content of alginate ≥10% *w/w*.

### 2.2. Characterization of the Composite Materials

**Figure 3:**
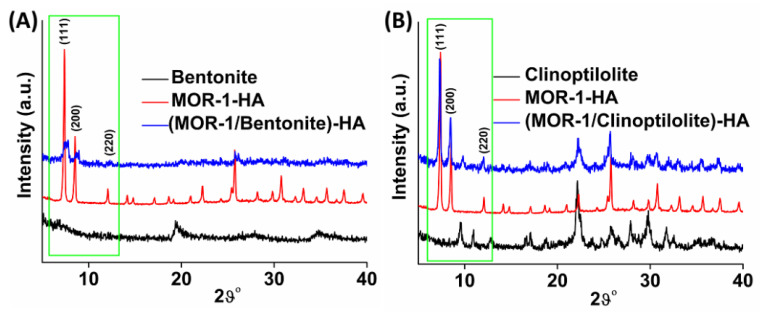
(**A**) PXRD patterns of bentonite, MOR-1-HA, and **(MOR-1/Bentonite)-HA.** (**B**) PXRD patterns of clinoptilolite, MOR-1-HA, and **(MOR-1/Clinoptilolite)-HA. The characteristic diffraction peaks of MOR-1 and aluminosilicates are highlighted in the green rectangles**.

In our previous work, we reported that MOR-1 and its alginic acid composite were highly crystalline materials [20,21]. The PXRD pattern of bentonite revealed a typical amorphous structure from 4 to 40 2θ (Figure 3A). In PXRD patterns obtained after the synthesis of **(MOR-1/Bentonite)-HA**, a slight loss of the crystallinity of MOR-1-HA was observed; however, the characteristic diffraction peaks of MOR-1-HA at 2θ of 7.3°, 8.5°, and 12° corresponding to (111), (200), and (220) planes, respectively, were present (Figure 3A). A typical PXRD pattern of clinoptilolite displays several diffraction peaks (Figure 3B) [31]. The PXRD pattern of **(MOR-1/Clinoptilolite)-HA** (Figure 3B) contained diffraction peaks of both MOR-1-HA and clinoptilolite, thus confirming the successful preparation of the composite material. Similarly, the PXRD patterns of Fe_3_O_4_-modified composites revealed great consistency with those of pristine materials (Figure S3).

**Figure 4:**
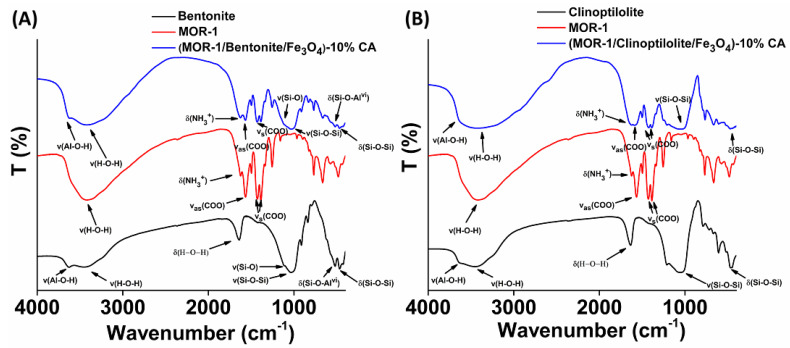
FT-IR spectra of (**A**) bentonite, MOR-1, and **(MOR-1/Bentonite)-HA**; (**B**) clinoptilolite, MOR-1, and **(MOR-1/Clinoptilolite)-HA**.

The successful modification of MOR-1 with either bentonite or clinoptilolite was further supported by FT-IR spectroscopy (Figure 4). The IR spectrum of **(MOR-1/Bentonite)-HA** indicated several characteristic bands of MOR-1, such as those at 1565 and 1383 cm^−1^ assigned to the ν*_as_*(COO^–^) and ν*_s_*(COO^–^) stretching modes, respectively, as well as the characteristic bands of pristine bentonite. Specifically, the peak at 3627 cm^−1^ was attributed to the vibration of the Al-O-H group, while the peaks at 1117 and 1030 cm^−1^ were due to the vibrations of Si-O units. Moreover, the bands at 524 and 463 cm^−1^ were related to the Al-O-Si and Si-O-Si bending vibrations of bentonite, respectively (Figure 4A) [32]. Similarly, the IR spectrum of **(MOR-1/Clinoptilolite)-HA** revealed the characteristic bands for MOR-1 as well as those for clinoptilolite (Figure 4B). In addition, the IR spectra of **(MOR-1/Bentonite/Fe_3_O_4_)-10%CA** and **(MOR-1/Clinoptilolite/Fe_3_O_4_)-10%CA** displayed the characteristic peaks for MOR-1 and either of those for bentonite (Figure S4a) or clinoptilolite materials (Figure S4b).

**Figure 5:**
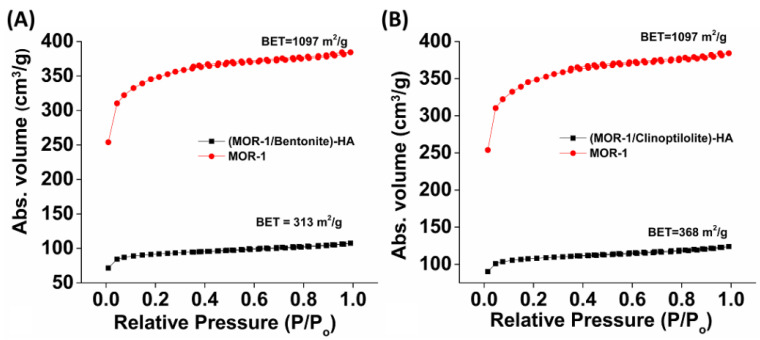
N_2_ sorption isotherms (77 K) for MOR-1, (**A**) **(MOR-1/Bentonite)-HA**, and (**B**) **(MOR-1/Clinoptilolite)-HA**.

Furthermore, diffuse reflectance UV-Vis data for the composite materials indicated absorption features of both MOR-1 and aluminosilicate components (Figures S5 and S6). The Brunauer–Emmett–Teller (BET) surface areas for **(MOR-1/Bentonite)-HA** and **(MOR-1/Clinoptilolite)-HA** were found to be 313 and 368 m^2^/g, respectively (Figure 5), whereas the BET surface areas of **(MOR-1/Bentonite/Fe_3_O_4_)-10%CA** and **(MOR-1/Clinoptilolite/Fe_3_O_4_)-10%CA** were determined to be 246 and 286 m^2^/g, respectively (Figure S7). In general, the surface areas of the composite materials were found to be significantly smaller than those of pristine MOR-1 (Figure 5), which implied a partial blockage of the pores of the MOF framework by the aluminosilicate material. Scanning electron microscopy (SEM) images revealed a two-phase morphology for all the composite materials (Figure S8). In addition, EDS analysis confirmed further the amalgamation of the MOF with bentonite or clinoptilolite (Figure S9), as well as the presence of Fe in the **(MOR-1/Bentonite/Fe_3_O_4_)-10%CA** and **(MOR-1/Clinoptilolite/Fe_3_O_4_)-10%CA** composite materials (Figures S10 and S11).

### 2.3. Batch Sorption Studies

#### 2.3.1. Sorption Kinetics 

**Figure 6:**
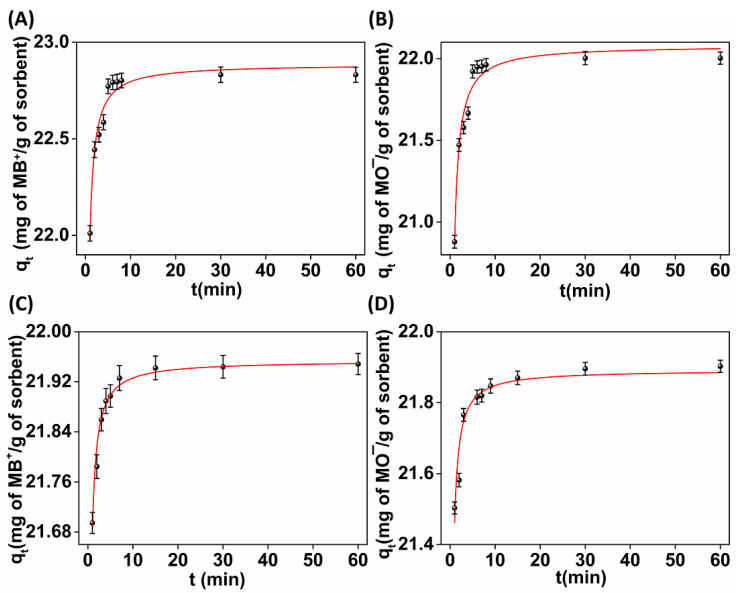
Fitting (red line) of the kinetics data with the Ho–Mckay’s pseudo-second-order equation for the sorption of (**A**) MB^+^ and (**B**) MO^−^ by **(MOR-1/Bentonite)-HA**; (**C**) MB^+^ and (**D**) MO^−^ by **(MOR-1/Clinoptilolite)-HA**.

The contact time was one of the critical factors that pointedly influenced the performance and assessed the applicability of the sorbent. Thus, the determination of the sorption kinetics was the first step in the investigation of the sorption properties of **(MOR-1/Bentonite)-HA** and **(MOR-1/Clinoptilolite)-HA** toward MB^+^ and MO^−^ ions. Lagergren’s first-order equation and Ho–Mckay’s pseudo-second-order equation are the commonly used models for the fitting of the kinetics data. In the current study, both models were applied; however, the significantly higher R^2^ values, the low values for the residual sum of squares and relatively small uncertainty parameters, suggested that Ho–Mckay’s pseudo-second-order equation better represented the kinetics of the dyes’ sorption (Table S1). This finding suggested a mechanism involving the chemisorption of dyes on the composite materials [33]. Figure 6 illustrates the effect of the contact time on the sorption of MB^+^ and MO^−^ by the composite materials.

The results indicated that the rate of sorption was very fast in the first 10 min of the interaction between the dyes’ ions and the sorbents. After that time, the removal rate became almost insignificant considering that by elapsing the contact time, the vacant sorption sites were less than in the beginning of the experiments. Significantly, within only the 1st min of contact, both materials successfully removed ~97% of the MO^−^ and ~98% of the MB^+^ ions. After 10 min of contact, an equilibrium was reached with removal percentages ≥ 99%.

#### 2.3.2. Sorption Isotherms

Equilibrium analysis is a powerful tool for the comprehension of the sorption process. Toward this end, we carried out sorption experiments with solutions of variable concentrations. The sorption equilibrium data were fitted with the Langmuir, Freundlich, and Langmuir−Freundlich isotherm models, the mathematical expressions of which are provided below:

(a) Langmuir
  $$q=q_{m}\frac{bC_{e}}{1+bC_{e}}$$
(b) Freundlich
  $$q=K_{F}C_{e}^{\frac{1}{n}}$$
(c) Langmuir–Freundlich

$$q=q_{m}\frac{{\left(bC_{e}\right)}^{\frac{1}{n}}}{1+{\left(bC_{e}\right)}^{\frac{1}{n}}}$$

where *q* (milligrams per gram) represents the amount of the ion removed at the equilibrium concentration *C_e_* (parts per million), *q_m_* is the maximum sorption capacity of the sorbent, *b* (milligrams per liter) is the Langmuir constant related to the free energy of the sorption, and *K_F_* and 1/*n* are the Freundlich constants [34].

**Figure 7:**
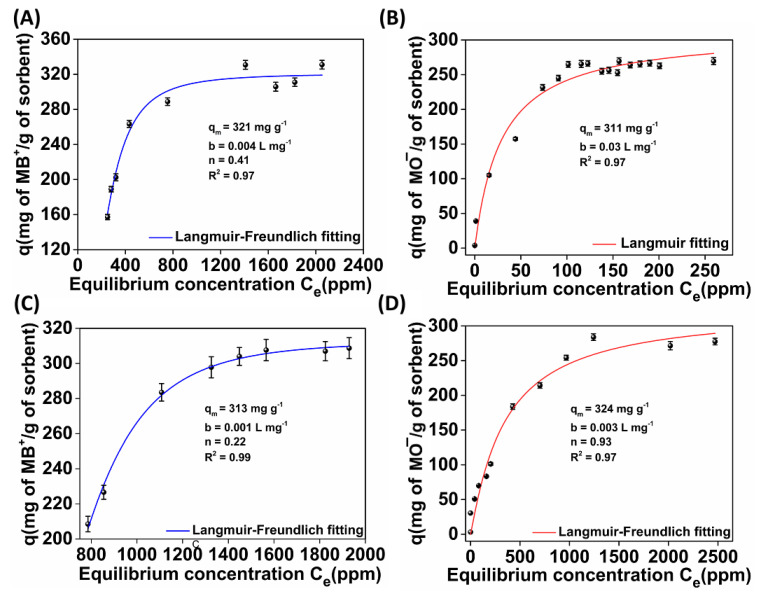
(**A**) MB^+^ and (**B**) MO^−^ isotherm sorption data for **(MOR-1/Bentonite)-HA**; (**C**) MB^+^ and (**D**) MO^−^ isotherm sorption data for **(MOR-1/Clinoptilolite)-HA**.

Figure 7 depicts the equilibrium data for the sorption of MB^+^ and MO^−^ ions by **(MOR-1/Bentonite)-HA** and **(MOR-1/Clinoptilolite)-HA,** as well as their fitting with the suitable model. The sorption isotherm data revealed that **(MOR-1/Bentonite)-HA** was able to remove 321 mg g^−1^ of MB^+^ and 312 mg g^−1^ of MO^−^ from the corresponding solutions. The best fitting of isotherm sorption data for **(MOR-1/Bentonite)-HA** was achieved with the Langmuir–Freundlich model for the sorption of MB^+^ and the Langmuir model for the sorption of MO^−^. The description of the MO^−^ sorption data by **(MOR-1/Bentonite)-HA** with the Langmuir model indicated a monolayer sorption of the dyes’ anionic species inside the pores of the MOR-1 material [35]. The fact that the MB^+^ sorption isotherm of **(MOR-1/Bentonite)-HA** followed the Langmuir–Freundlich model, which combines features of both the Langmuir and Freundlich approaches, indicated the contribution of both homogenous and heterogenous sorption processes [35]. In our previous study, we reported that the sorption of MO^−^ by MOR-1-HA was attributed to the exchange of Cl^−^ ions with MO^−^ ions. However, no sorption capacity was observed for the cationic dye MB^+^, which has a similar molecular size to MO^−^, revealing that MOR-1–HA material can selectively sorb the anionic but not the cationic dye through an ion-exchange process [12]. These findings suggested that bentonite was responsible for the removal of MB^+^ ions and not the MOR-1-HA. Indeed, the latter was supported by the equilibrium data obtained from the sorption of MB^+^ by the pristine bentonite material. The maximum sorption capacity was found to be 525 mg g^−1^ (Figure S12a). The value of 321 mg of MB^+^ removed per gram of **(MOR-1/Bentonite)-HA** was close to the theoretical, given that the weight percentage of bentonite in the composite material was 50%. 

The isotherm sorption data for **(MOR-1/Clinoptilolite)-HA** were fitted with the Langmuir–Freundlich model for both the MB^+^ and MO^−^ sorption processes. The maximum MB^+^ and MO^−^ sorption capacities for **(MOR-1/Clinoptilolite)-HA** were calculated to be 312 mg g^−1^ and 323 mg g^−1^, respectively. Pristine clinoptilolite was able to remove 403 mg of ΜΒ^+^ g^−1^ (Figure S12b), while the composite **(MOR-1/Clinoptilolite)-HA** seemed to be more effective for the removal of MB^+^. It is worth mentioning that bentonite and clinoptilolite showed no sorption capacity for MO^−^. The results acquired from the isotherm studies in combination with those previously obtained for MOR-1-HA [12] revealed that the sorption process involved the cation exchange of MB^+^ in the aluminosilicate component and anion exchange of MO^−^ in the MOR-1 material. 

#### 2.3.3. Variable pH Studies

**Figure 8:**
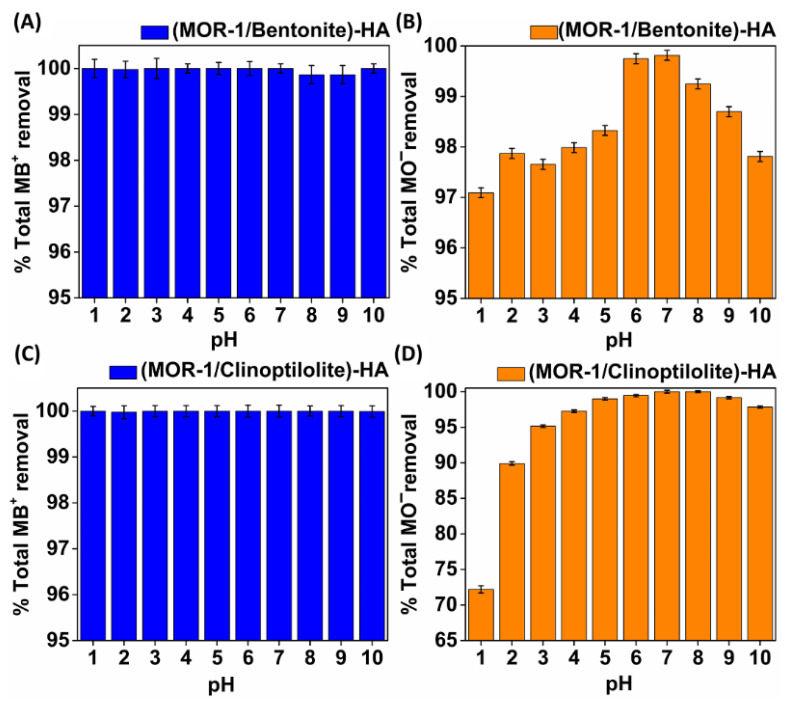
Percentage of sorption of (**A**) MB^+^ and (**B**) MO^−^ by **(MOR-1/Bentonite)-HA**; (**C**) MB^+^ and (**D**) MO^−^ by **(MOR-1/Clinoptilolite)-HA** in the pH range of 1–10.

Detailed studies with dye-contaminated solutions of a wide pH range revealed that pH was not a critical factor for the performance of our sorbents. The pH study was conducted with solutions containing MB^+^ or MO^−^ and in the pH range from 1 to 10 for both materials. Significantly, the capture of MB^+^ from either **(MOR-1/Bentonite)-HA** or **(MOR-1/Clinoptilolite)-HA** was overwhelming since the removal percentages were close to 100% (>99.86–100%) independent of the pH of the solution (Figure 8A,C). Furthermore, **(MOR-1/Bentonite)-HA** exhibited astonishing sorptive behavior toward the anionic dye MO^−^, with removal percentages higher than 97% (Figure 8B). **(MOR-1/Clinoptilolite)-HA** was found capable to remove MO^−^ as effectively as **(MOR-1/Bentonite)-HA** in solutions with a pH > 3, while a slight loss was observed at pH 2 and 1, with removal percentages reaching 90% and 72% (Figure 8D), respectively. It is likely that the high excess of Cl^−^ anions resulted from the HCl acid used for pH adjustment and not that the presence of H^+^ affected the sorption of MO^−^ by (**MOR-1/Clinoptilolite)-HA**. It is not clear, however, why the sorption of MO^−^ by **(MOR-1/bentonite)-HA** was inhibited only a little under similar conditions.

#### 2.3.4. Selectivity Studies

**Figure 9:**
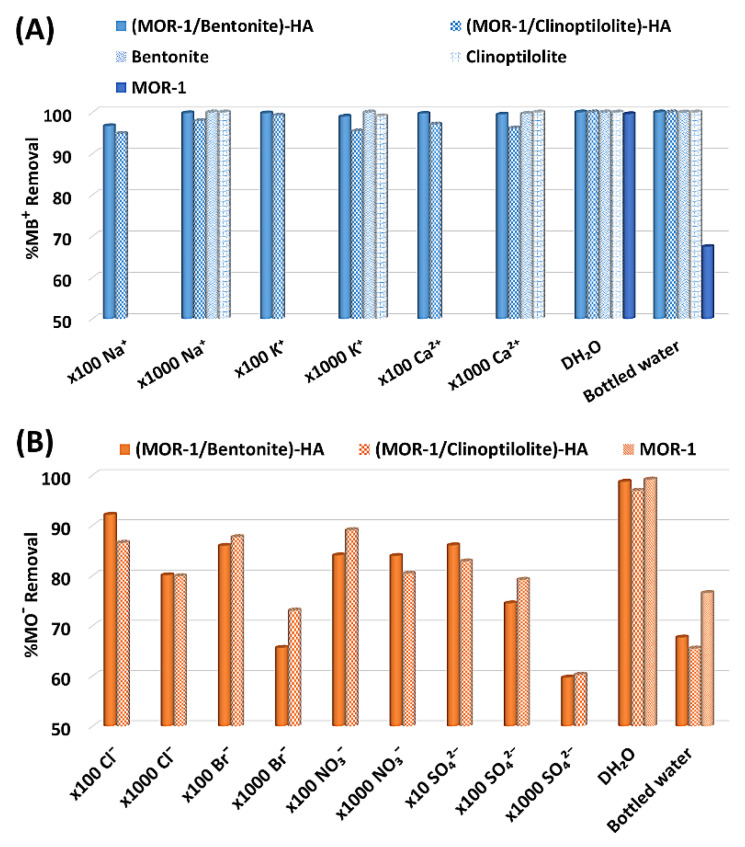
(**A**) MB^+^ sorption data for **(MOR-1/Bentonite)-HA** and **(MOR-1/Clinoptilolite)-HA** in the presence of various competitive anions (initial MB^+^ concentration = 18 ppm, pH ∼ 6.5) and in contaminated bottled water samples (initial MB^+^ concentration = 18 ppm, pH ∼ 7.8). For comparison, sorption results in distilled water (DH_2_O) solutions (containing no antagonistic ions) are also provided. (**B**) MO^−^ sorption data for **(MOR-1/Bentonite)-HA** and **(MOR-1/Clinoptilolite)-HA** in the presence of various competitive anions (initial MB^+^ concentration = 18 ppm, pH ∼ 6.5), in contaminated bottled water samples (initial MB^+^ concentration = 19 ppm, pH ∼ 7.8), and in distilled water solutions. The composition of the bottled water was as follows: pH = 7.8, HCO_3_^−^ = 244 ppm, Cl^−^ = 4.29 ppm, NO_3_^−^ = 1.93 ppm, SO_4_^2−^ = 9.16 ppm, Na^+^ = 2.24 ppm, K^+^ = 0.6 ppm, Ca^2+^ = 80.7 ppm, and Mg^2+^ = 5.34 ppm.

Given the fact that dye-contaminated wastewater contains several anionic and/or cationic species besides the dyes’ ions, dye sorption studies were also performed in complex solutions with a number of antagonistic cations and/or anions. To this end, the sorptive ability of **(MOR-1/Bentonite)-HA** and **(MOR-1/Clinoptilolite)-HA** toward MB^+^ was investigated in solutions containing a series of competitive cations, such as Na^+^, K^+^, and Ca^2+^ in large excesses. Interestingly, the MB^+^ removal ability of both composites seemed not to be importantly influenced by the presence of the antagonistic cations. In particular, **(MOR-1/Bentonite)-HA** achieved removal percentages for MB^+^ as high as 99.80%, 98.95%, and 99.46% even in the presence of a 1000-fold excess of Na^+^, K^+^, and Ca^2+^, respectively, while the correspondent percentages for **(MOR-1/Clinoptilolite)-HA** were found to be 97.90%, 95.40%, and 96.11% (Figure 9A). In addition, the ability of **(MOR-1/Bentonite)-HA** and **(MOR-1/Clinoptilolite)-HA** to capture MO^−^ was investigated in aqueous solutions containing Cl^−^, Br^−^, NO_3_^−^, or SO_4_^2−^ anions in relatively high concentrations. As shown in Figure 9B, both composite materials could efficiently remove MO^−^, even in the presence of quite high concentrations of competitive anionic species. Specifically, despite the presence of 1000-fold excesses of Cl^−^ or NO_3_^−^, the removal percentages of MO^−^ by **(MOR-1/Bentonite)-HA** and **(MOR-1/Clinoptilolite)-HA** were calculated to be equal or higher than 80%. A slight decrease was observed when a 1000-fold excess of Br^−^ was added to the solutions (removal percentages = 65.6% and 73.0% for **(MOR-1/Bentonite)-HA** and **(MOR-1/Clinoptilolite)-HA,** respectively), and even higher was the decrease after adding a 1000-fold of SO_4_^2−^ (~60% removal). However, the latter finding can be easily explained since SO_4_^2−^ is bivalent and can interact more efficiently with the MOF’s active sites than the monoanionic MO^−^. The last step to the selectivity study was the investigation of the efficiency of **(MOR-1/Bentonite)-HA** and **(MOR-1/Clinoptilolite)-HA** to uptake the ions of dyes under realistic conditions. For this reason, the sorption occurred in bottled water intentionally contaminated with MB^+^ or MO^−^. These samples were rich in several anionic and cationic species, including Cl^−^, NO_3_^−^, SO_4_^2−^, HCO_3_^−^, Na^+^, K^+^, Ca^2+^, and Mg^2+^, with concentrations that exceeded those of MB^+^ and MO^−^ by up to 35 times. Although the removal of MO^−^ seemed to be slightly hindered due to the mixture of the anions, the removal percentages of 67.64% and 65.45% with **(MOR-1/Bentonite)-HA** and **(MOR-1/Clinoptilolite)-HA** were still high and very promising, considering the complexity of these solutions (Figure 9). Moreover, the selective removal of MB^+^ by both **(MOR-1/Bentonite)-HA** and **(MOR-1/Clinoptilolite)-HA** was exceptional, since the presence of the ions had zero effect on the sorption process and no traces of the dyes were detected in the bottled samples. 

### 2.4. Column Sorption Study 

The above sorption results derived from the batch reaction experiments are promising for the possible use of **(MOR-1/Bentonite)-HA** and **(MOR-1/Clinoptilolite)-HA** in MO^−^ and MB^+^ sorption applications. However, the efficient performance of ion-exchange materials under stirring conditions does not necessarily mean that they can be applied in industrial wastewater treatment. The latter requires the use of continuous flow ion-exchange columns [19]. Furthermore, dye-contaminated industrial wastewater contains more than one coloring factor, which can be either anionic or cationic. With a view to address this issue, we decided to perform column sorption studies with a mixture of MB^+^/MO^−^, which better met the requirements for real-world wastewater treatment. As reported above, **(MOR-1/Bentonite)-HA** and **(MOR-1/Clinoptilolite)-HA** are in the form of fine powder, and thus they cannot be used as stationary phases in columns for the removal of dyes under continuous flow. Thus, two new composite materials in the form of beads were isolated and used for the simultaneous removal of a mixture of MB^+^/MO^−^ under dynamic conditions, namely **(MOR-1/Bentonite/Fe_3_O_4_)-10%CA** and **(ΜOR-1/Clinoptilolite/Fe_3_O_4_)-10%CA**. Column sorption studies were conducted with a MB^+^/MO^−^ mixture solution of an initial concentration of 3.7 ppm MB^+^ and 5.3 ppm MO^+^. Since the light green solution passed through the column, it was decolorized (Figure S13). Importantly, the columns could be easily regenerated by treatment with a solution of 1M HCl and reused for several cycles. Specifically, a column filled with **(MOR-1/Bentonite/Fe_3_O_4_)-10%CA beads** achieved the removal of 82% of MB^+^ from the first bed volume received (bed volume = bed height [cm] x cross sectional area [cm^2^]), while after 115 bed volumes, the correspondent removal percentage was close to 48% of the initial MB^+^ concentration (Figure S14a). At the same time, 69% and 20% removal were observed for MO^−^ at the first and last bed volumes collected, respectively (Figure S14b). In the second and third runs, the performance of the column seemed to be improved due to the slower flow rate, which resulted in a longer contact time. The bed volume was 1.15 mL, the average flow rate = 1.66 mL min^−1^, and the empty bed contact time (EBCT) = 0.69 min [36]. Specifically, in the first bed volume collected in the second run, the removal percentages were increased to 88% and 86% for MB^+^ and MO^−^, respectively, whereas the corresponding percentages found in the third run of the column were as high as 96% and 90%, respectively (Figure S14). It is worth mentioning that 83% and 75% removal of the initial MB^+^ and MO^−^ content, respectively, could be achieved even after passing 85 bed volumes in the third run of the column. Similar results were obtained from the ion-exchange column filled with **(ΜOR-1/Clinoptilolite/Fe_3_O_4_)-10%CA** beads (bed volume = 1.34 mL, the average flow rate = 2.5 mL min^−1^, and the EBCT = 0.54 min) (Figure S15)_._

**Figure 10:**
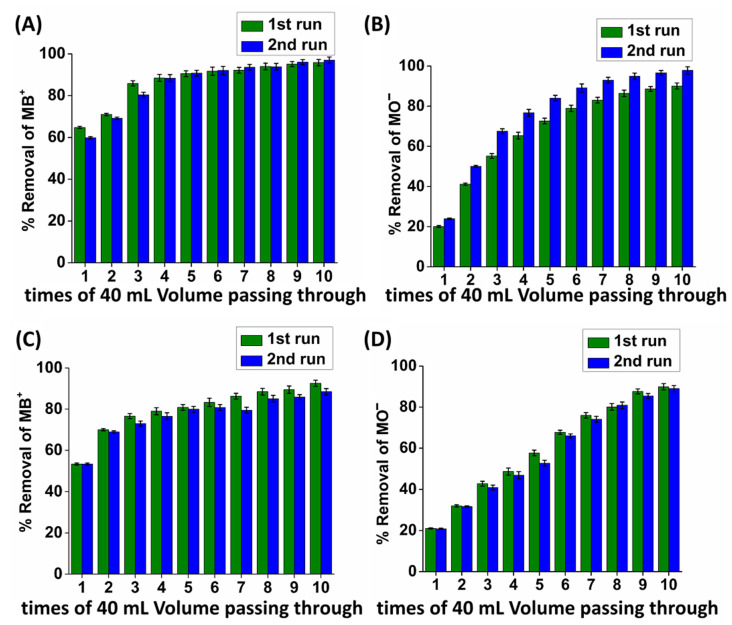
Column sorption data regarding the removal of (**A**) MB^+^ and (**B**) MO^−^ with **(MOR-1/Bentonite/Fe_3_O_4_)-10%CA** from the mixture solution. Column sorption data regarding the removal of (**C**) MB^+^ and (**D**) MO^−^ with **(ΜOR-1/Clinoptilolite/Fe_3_O_4_)-10%CA** from the mixture solution. The MB^+^/MO^−^ mixture solution circularly passed through the column up to 10 times.

Furthermore, we designed a column sorption investigation where 40 mL of the mixture solution circularly passed through the column. The latter simulated a popular procedure for industrial wastewater treatment, where a series of columns was applied to improve the removal performance of the sorbent [19]. The results proved that both types of columns were impressive, since both **(MOR-1/Bentonite/Fe_3_O_4_)-10%CA** and **(ΜOR-1/Clinoptilolite/Fe_3_O_4_)-10%CA** were able to successfully capture approximately 90% of the initial MB^+/^MO^−^ content (Figure 10). Specifically, **(MOR-1/Bentonite/Fe_3_O_4_)-10%CA** was able to remove 95% and 92% of the MB^+^ and MO^−^, respectively, after 10 times of circularly passing the effluent through the column (bed volume = 1.15 mL, the average flow rate = 8 mL min^−1^, and the EBCT = 0.14 min) (Figure 10A,B). It should be noted that the column was treated with 1M HCl and was reused showing a similar removal capacity. Likewise, the removal percentages for **(ΜOR-1/Clinoptilolite/Fe_3_O_4_)-10%CA** were as high as 96% and 90% for MB^+^ and MO^−^, respectively (bed volume = 1.34 mL, the average flow rate = 8 mL min^−1^, and the EBCT = 0.17 min) (Figure 10C,D). The regeneration and reuse of this column revealed slight changes in the removal capability of the column. Noteworthily, the average flow rate of the columns was calculated to be 8 mL min^−1^, which meant that 5 min was enough to complete every cycle of 40 mL solution feeding. The latter finding is very promising for industrial applications, not only because a single column with circularity feeding would be able to sufficiently downgrade a mixture of dye-contaminated wastewater, but also due to the short operation time and the “ready to use” ability after a simple regeneration procedure. In addition, the regeneration of the composite materials was achieved inside the column, in contrast to the traditional mixed bed columns where the regeneration demands removal and repacking at the stationary phase [19].

Although we cannot clarify if a synergistic effect occurred in the simultaneous sorption of anionic and cationic dyes, several findings from this study supported the opposite scenario. The batch sorption data with solutions containing either MB^+^ or MO^−^ (individual dye sorption experiments) indicated in general the higher sorption capability of composites for MB^+^ vs. MO^−^. This trend seemed to be followed also in column sorption with a mixture of dyes, and thus synergistic sorption phenomena were not rather likely.

### 2.5. Isolation and Characterization of the Composite Materials and Dye-Loaded Composite Materials

**Figure 11:**
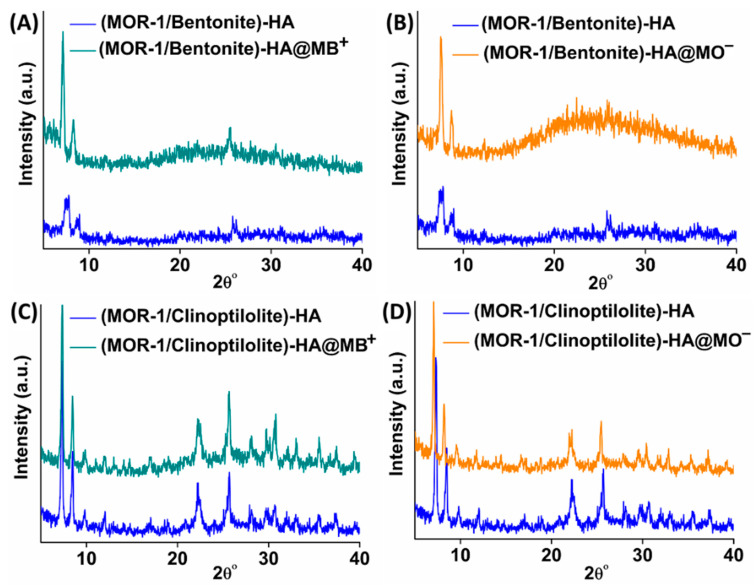
PXRD patterns of **(MOR-1/Bentonite)-HA** along with those of (**A**) **(MOR-1/Bentonite)-HA@MB^+^**, (**B**) **(MOR-1/Bentonite)-HA@MO^−^** and **MOR-1/Clinoptilolite)-HA**, (**C**) **(MOR-1/Clinoptilolite)-HA@MB^+^**, and (**D**) (**MOR-1/Clinoptilolite)-HA@MO^−^**.

The color of both composite materials changed from light yellow to blue or orange after the sorption of MB^+^ or MO^−^, respectively (Figures S16 and S17). Moreover, UV−vis diffuse reflectance spectroscopy further supported the capture of the dyes’ ions by the composite materials. Specifically, the wide band appearing in the region of 550 to 800 nm at the spectra of **(MOR-1/Bentonite)-HA@MB^+^** (Figure S17a) and **(MOR-1/Clinoptilolite)-HA@MB^+^** (Figure S19a) was assigned to the absorption of MB^+^. Similarly, in Figures S18b and S19b, the characteristic peak at 464 nm was due to the absorption of MO^−^. In addition, UV-vis spectra of the materials used as the stationary phase in the ion-exchange columns displayed absorption peaks of both MB^+^ and MO^−^, confirming the simultaneous sorption of the two anionic species by the composites (Figure S18). The PXRD patterns of **(MOR-1/Bentonite)-HA@MB^+^, (MOR-1/Bentonite)-HA@MO^−^, (MOR-1/Clinoptilolite)-HA@MB^+^,** and **(MOR-1/Clinoptilolite)-HA@MO^−^** revealed that the crystal structures were retained after the sorption processes (Figure 11). Moreover, the PXRD patterns obtained from **(MOR-1/Bentonite/Fe_3_O_4_)-10%CA** and **(MOR-1/Clinoptilolite/Fe_3_O_4_)-10%CA beads**, after the treatment of the column with the mixture of MB^+^ and MO^−^, indicated that the crystallinity of the composites was preserved (Figure S21). 

## 3. Conclusions

In conclusion, this study dealt with the development of novel composite sorbents with the capability for the simultaneous removal of anionic and cationic toxic dyes from aqueous media. Specifically, the two composite materials, **(MOR-1/Bentonite)-HA** and **(MOR-1/Clinoptilolite)-HA**, were synthesized via a facile method and their sorptive efficiencies toward the toxic dyes MB and MO were investigated in detail. **(MOR-1/Bentonite)-HA** and **(MOR-1/Clinoptilolite)-HA** exhibited a high sorption capacity, fast sorption kinetics (the equilibrium can be achieved in ~10 min), excellent sorption ability in acidic and alkaline solutions, and high selectivity for the dyes over various coexisting ionic species. Toward practical applications, **(MOR-1/Bentonite/Fe_3_O_4_)-10%CA** and **(MOR-1/Clinoptilolite/Fe_3_O_4_)-10%CA** in the form of beads were used as stationary phases in columns, achieving the highly efficient removal of MB^+^ and MO^−^ ions from a mixture containing both dyes. Overall, this study provides an alternative technology combining the high removal efficiency of multiple pollutants and capability for the in-situ regeneration of sorbents, as opposed to well-known mixed bed columns requiring the ex-situ regeneration of resins, a process that causes significant delays to the water treatment procedure and increased costs.

## Data Availability

Not applicable.

## Supplementary Materials

The supporting information can be downloaded at: https://www.mdpi.com/article/10.3390/molecules28020815/s1, Figure S1: Representation of the structures of bentonite and clinoptilolite; Figure S2: Optical image of (MOR-1/Bentonite/Fe_3_O_4_)-10%CA beads and recovery of the (MOR-1/Bentonite/Fe_3_O_4_)-10%CA beads by applying magnetic field; Figure S3: PXRD patterns of bentonite, MOR-1-HA, (MOR-1/Bentonite/Fe_3_O_4_)-10%CA and clinoptilolite, MOR-1-HA, (MOR-1/Clinoptilolite/Fe_3_O_4_)-10%CA; Figure S4: FTIR spectra of bentonite, MOR-1, (MOR-1/Bentonite/Fe_3_O_4_)-10%CA and clinoptilolite, MOR-1, (MOR-1/Clinoptilolite/Fe_3_O_4_)-10%CA; Figure S5: Solid state UV spectra of bentonite, MOR-1, (MOR-1/Bentonite)-HA and Clinoptilolite, MOR-1, (MOR-1/Clinoptilolite)-HA; Figure S6: Solid state UV spectra of bentonite, MOR-1, (MOR-1/Bentonite/Fe_3_O_4_)-10%CA and clinoptilolite, MOR-1, (MOR-1/Clinoptilolite/Fe_3_O_4_)-10%CA; Figure S7: N_2_ sorption isotherms (77 K) for MOR-1, (MOR-1/Bentonite/Fe_3_O_4_)-10%CA and (MOR-1/Clinoptilolite/Fe_3_O_4_)-10%CA; Figure S8: SEM images of (MOR-1/Bentonite)-HA, (MOR-1/Clinoptilolite)-HA, (MOR-1/Bentonite/Fe_3_O_4_)-10%CA bead and (MOR-1/Clinoptilolite/Fe_3_O_4_)-10%CA; Figure S9: EDS spectra of (MOR-1/Bentonite)-HA and (MOR-1/Clinoptilolite)-HA; Figure S10: EDS spectrum of (MOR-1/Bentonite/Fe_3_O_4_)-10%CA; Figure S11: EDS spectrum of (MOR-1/Clinoptilolite/Fe_3_O_4_)-10%CA; Figure S12: MB^+^ isotherm sorption data for bentonite and clinoptilolite; Figure S13: MB^+^/MO^-^ mixture solution, the colorless effluent of the column and UV spectra of the MB^+^/MO^-^ mixture solution; Figure S14: Column sorption data for (MOR-1/Bentonite/Fe_3_O_4_)-10%CA beads; Figure S15: Column sorption data for (MOR-1/Clinoptilolite/Fe_3_O_4_)-10%CA beads; Figure S16: Optical images of (MOR-1/Bentonite)-HA, (MOR-1/Bentonite)-HA@MB^+^ and (MOR-1/Bentonite)-HA@MO^-^; Figure S17: Optical images of (MOR-1/Clinoptilolite)-HA, (MOR-1/Clinoptilolite)-HA@MB^+^ and (MOR-1/ Clinoptilolite)-HA@MO^-^; Figure S18: Solid state UV spectra of (MOR-1/Bentonite)-HA in comparison with (MOR-1/Bentonite)-HA@MB^+^ and (MOR-1/Bentonite)-HA@MO^-^; Figure S19: Solid state UV spectra of (MOR-1/Clinoptilolite)-HA in comparison with (MOR-1/ Clinoptilolite)-HA@MB^+^ and (MOR-1/ Clinoptilolite)-HA@MO^-^; Figure S20: Solid state UV spectra of (MOR-1/Bentonite/Fe_3_O_4_)-10%CA and (MOR-1/Clinoptilolite/Fe_3_O_4_)-10%CA, before and after the sorption; Figure S21: PXRD patterns of (MOR-1/Bentonite/Fe_3_O_4_)-10%CA and (MOR-1/Clinoptilolite/Fe_3_O_4_)-10%CA, before and after the sorption; Table S1: The fitting parameters for kinetics. References [37,38,39,40,41,42,43] are cited in the Supplementary Materials.

## Sample Availability

Samples of the compounds are available from the authors.
