# Supplementary material for: Composite Materials Based on a Zr4+ MOF and Aluminosilicates for the Simultaneous Removal of Cationic and Anionic Dyes from Aqueous Media
Source: Molecules. 2023 Jan 13;28(2):815. doi: 10.3390/molecules28020815 (PMC9864044; doi:10.3390/molecules28020815)
Supplement: Supplementary file 1 [file molecules-28-00815-s001.zip › molecules-2097107-supplementary.pdf]

molecules

MDPI

Supplementary Information

Composite Materials Based on a Zr^{4+} MOF and Aluminosilicates for the Simultaneous Removal of Cationic and Anionic Dyes from Aqueous Media

Petros Georgianos [1], Anastasia D. Pournara [1], Evangelos K. Andreou [2], Gerasimos S. Armatas [2] and Manolis J. Manos [1,3,*]

1 Department of Chemistry, University of Ioannina, GR-45110 Ioannina, Greece
2 Department of Materials Science and Technology, University of Crete, GR-70013 Heraklion, Greece
3 Institute of Materials Science and Computing, University Research Center of Ioannina, GR-45110 Ioannina, Greece
* Correspondence: emanos@uoi.gr

Table of Contents

EXPERIMENTAL SECTION S2

CHEMICALS S2
SYNTHESIS S2
PREPARATION OF (MOR-1/BENTONITE/FE_3O_4)-10% CALCIUM ALGINATE BEADS S2
SORPTION STUDIES S2
COLUMN SORPTION STUDY S3
CHARACTERIZATION TECHNIQUES S3

RESULTS AND DISCUSSION S4

SYNTHESIS OF THE COMPOSITE MATERIALS S4
CHARACTERIZATION OF THE COMPOSITE MATERIALS S5
UV-VIS CHARACTERIZATION S5
BATCH ION-EXCHANGE STUDIES S8

REFERENCES S12

Experimental Section

Chemicals

All the chemicals were commercially available and used without further purification. Zirconium chloride ($ZrCl_4$), 2-aminoterephthalic (NH_2-H_2BDC) and $FeCl_3.6H_2O$ were purchased from Aldrich. Natural bentonite clay and clinoptilolite were purchased from commercial sources. Sodium alginate powder was purchased from Aldrich and used as received. The solvents were used as received.

Synthesis

The synthesis and characterization of **MOR-1** and **MOR-1-HA** was reported previously by us.[20,21]

Synthesis of (MOR-1/Bentonite)-HA and (MOR-1/Clinoptilolite)-HA

A suspension of **(MOR-1/Bentonite)-SA** or **(MOR-1/Clinoptilolite)-SA)** was prepared by dispersing 0.1 g MOR-1 and 0.1g bentonite (or 0.1g clinoptilolite) in 7.85 mL 50% w/v sodium alginate aqueous solution via ultrasonication. The mixture was kept under stirring for 30 min. To this suspension, CH_3COOH was added dropwise, resulting to the formation of the composite materials **(MOR-1/Bentonite)-HA** and **(MOR-1/Clinoptilolite)-HA.** CH_3COOH converts sodium alginate to alginic acid which is insoluble in water and encapsulates the sorbent particles forming a thin layer around them.[2] The composite materials were isolated via filtration using a Buchner funnel, washed several times with excess of water to remove the spare amount of unbound calcium, and dried overnight at 60 °C (Yield: 0.146 g of **(MOR-1/Bentonite)-HA**) and 0.170 g of **(MOR-1/Clinoptilolite)-HA**.

Preparation of (MOR-1/Bentonite/Fe_3O_4)-10% calcium alginate beads

In a suspension of 0.255 g MOR-1 and 0.255 g bentonite in 10 mL distilled water, 0.03 g Fe_3O_4 were added. The mixture was kept under stirring for 30 min. To this suspension, 0.06 g of sodium alginate were added, and the mixture was stirred vigorously for 1-2 h. The resulting suspension was dropped through a syringe needle into 2% (w/v) $CaCl_2$ solution where the spherization was performed. In order to improve the mechanical strength of the beads, they were kept in the mother liquor for 30 min. Afterwards, the isolation of the fully formed spheres occurred via filtration. The beads were washed with excess of water to remove any unbound amount of calcium and dried overnight at 60 °C (Yield: 0.506 g).

The later procedure was also followed for the preparation of (MOR-1/Clinoptilolite/Fe_3O_4)-10% calcium alginate beads (Yield: 0.512 g), with the exception that Clinoptilolite was used instead of Bentonite.

Sorption studies

Preparation of the column. 0.6 g of **(MOR-1/Bentonite/Fe_3O_4)-10%CA beads** were used to fill a glass column (0.7 cm ID column). Prior the sorption study, the column was washed with ~ 50 mL HCl (1 M) solution, in order to protonate the amine groups and 200 mL deionized water to remove the excess of HCl. A second column was filled up with 0.6 g of **(MOR-1/Clinoptilolite/Fe_3O_4)-10%CA beads** and was treated as above.

Batch sorption studies. A typical sorption experiment of the composite material **(MOR-1/Bentonite)-HA** (or **(MOR-1/Clinoptilolite)-HA**) with the MB^+ or MO^- is the following: In a solution of MB^+ or MO^- (0.4 mmol) in water (10 mL, pH ~ 7), 0.04 mmol of **(MOR-1/Bentonite)-HA** (or **(MOR-1/Clinoptilolite)-HA**) was added as a solid. The mixture was kept under magnetic stirring for ~ 1 h. Then, the solid material was isolated by filtration, washed several times with water and acetone and dried in the air. The MB^+ or MO^- uptake from solutions of various concentrations was studied by the batch method

at V:m ~ 1000 mL/g, room temperature and 1 h of contact. The competitive and variable pH ion exchange experiments were also carried out with the batch method at V: m ratio (1000) mL/g, room temperature and 1 h contact. For the determination of the sorption kinetics, MB^+ or MO^- ion-exchange experiments of various reaction times (1-60 min) have been performed. For each experiment, a 10 mL sample of MB^+ or MO^- solution (initial concentration = 21.6 ppm, pH~7) was added to each vial and the mixtures were kept under magnetic stirring for the designated reaction times. The suspensions from the various reactions were filtrated and the resulting solutions were analyzed for their MB^+ or MO^- content with UV-Vis spectroscopy. Each sorption experiment has been done at least twice and the reported sorption data represent the average of sorption results from the different sorption experiments. The difference between the concentrations of the dyes determined for the different sorption experiments was <2%. Batch MO^- and MB^+ sorption tests with calcium alginate (CA) beads (containing no sorbent material) indicated no sorption capacity for the dyes.

Column Sorption Study

Column ion-exchange study. 1st Procedure: An aqueous solution containing a mixture of MB^+ (3.7 ppm) and MO^- (5.3 ppm) was prepared. The mixture was passed through the column and the effluents were collected in vials and analyzed via UV-vis spectroscopy. For the regeneration of the column ~ 50 mL of HCl acid (1 M) solution. Then, the column was washed with 200 mL of water to remove excess acid and tested again for MB^+/MO^- simultaneous ion-exchange. The column was regenerated and reused for two cycles after the first application. Column sorption study with calcium alginate beads showed no removal capacity for MB^+ or MO^- ions. **2nd Procedure:** 40mL of an aqueous solution containing a mixture of MB^+ (3.7 ppm) and MO^- (5.3 ppm) was prepared. The mixture was passed through the column and the effluent was collected in a flask and analyzed via UV-vis spectroscopy. The effluent was circularly passed through the column and analyzed up to 10 consecutive times. Subsequently, the column was regenerated with ~ 50 mL of 1M HCl, washed with 200 mL of water to remove excess acid and tested again for MB^+/MO^- simultaneous ion-exchange as above.

Characterization Techniques

Powder X-ray diffraction patterns were recorded on a Bruker D8 Advance X-ray diffractometer (CuK_a radiation, λ=1.5418 Å). IR spectra were recorded in the range of 4000-400 cm^{-1} range using an Agilent Cary 630 FT-IR. UV/vis MB^+ and MO^- solution spectra were obtained on an Agilent Cary 4000 in the wavelength range of 200-800 nm. UV−vis diffuse reflectance spectra were also obtained on an Agilent Cary 4000. Images and Energy dispersive spectroscopy (EDS) analyses were recorded on a JEOL JSM-6390LV scanning electron microscope (SEM) equipped with an Oxford INCA PentaFET-x3 energy dispersive X-ray spectroscopy (EDS) detector. N_2 adsorption-desorption isotherms were measured at 77K on a Quantachrome Nova 3200e sorption analyzer. Before analysis, all samples were EtOH exchanged, activated via supercritical CO_2 drying and then, degassed at 150 ^{0}C under vacuum (<10^{-5} Torr) for 12 h. The specific surface areas were calculated by applying the Brumauer-Emmett-Teller (BET) method to the absorption branch of isotherms in the 0.04–0.23 relative pressure (P/P_o) range.

Results and discussion

Synthesis of the composite materials

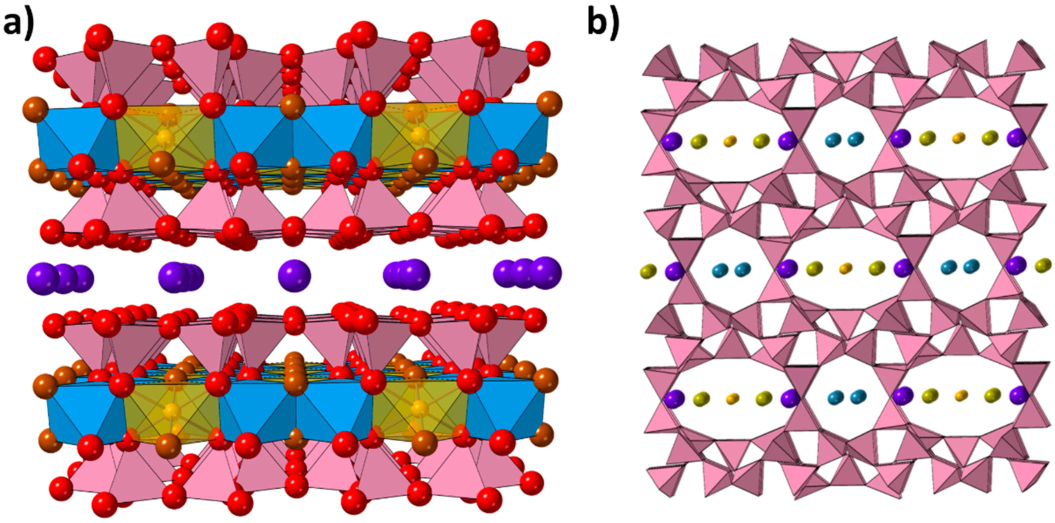

Figure S1. Representation of the structures of (**a**) bentonite and (**b**) clinoptilolite. Bentonite is mainly (80%) made from montmorillonite, a clay mineral with the general formula $K_{0.58}(Al,Mg)(Si,Al)O_{10}(OH)_2$. Clinoptilolite is a zeolite mineral with the general formula $(K, Na, Ca)_6[(Si, Al)_{36}O_{72}]\ nH_2O$ (n = 20–24). Color code: Al, blue; K, purple; Mg, dark yellow; O, red; OH, brown; Si, pink; Ca, turquoise; Na, light yellow.

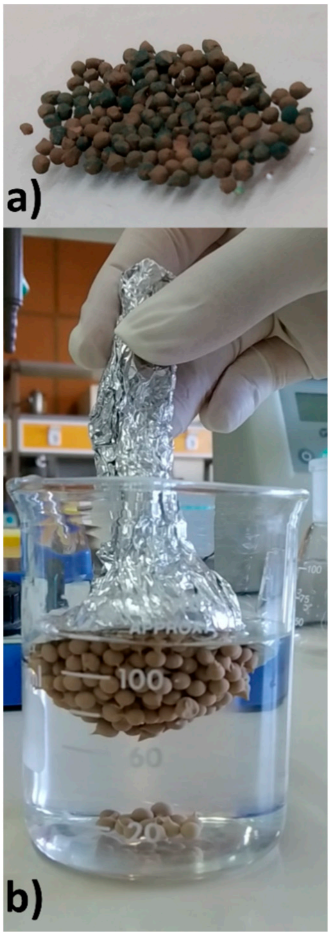

Figure S2. (**a**) Optical image of (MOR-1/Bentonite/Fe_3O_4)-10%CA beads, (**b**) Recovery of the (MOR-1/Bentonite/Fe_3O_4)-10%CA beads by applying magnetic field.

Characterization of the composite materials

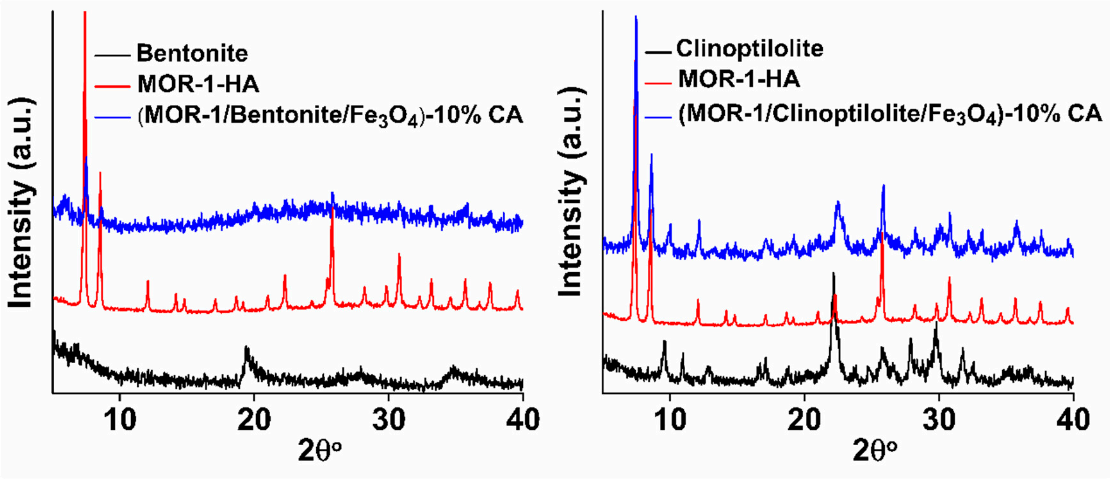

Figure S3. PXRD patterns of (**a**) bentonite, MOR-1-HA, (MOR-1/Bentonite/Fe_3O_4)-10%CA and (**b**) clinoptilolite, MOR-1-HA, (MOR-1/Clinoptilolite/Fe_3O_4)-10%CA.

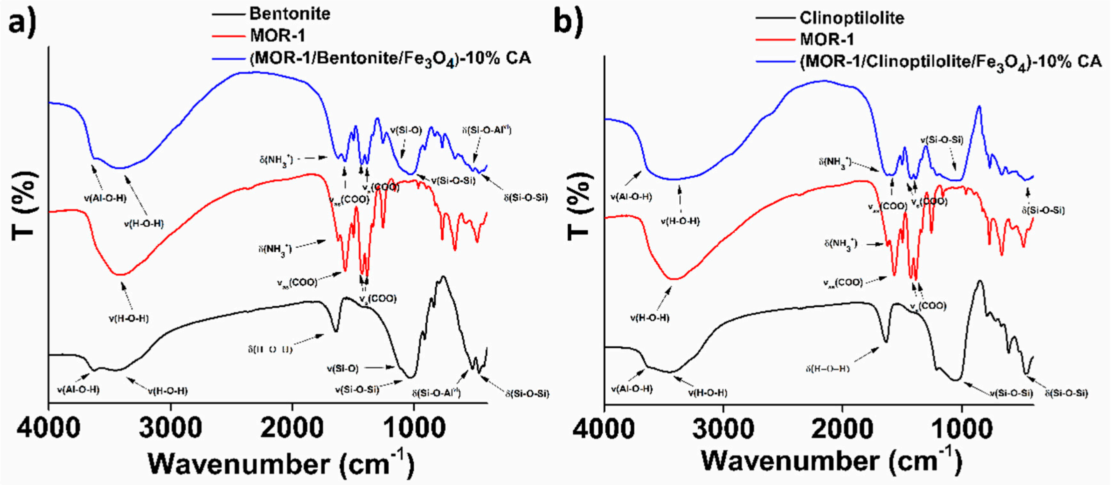

Figure S4. FTIR spectra of (**a**) bentonite, MOR-1, (MOR-1/Bentonite/Fe_3O_4)-10%CA and (**b**) clinoptilolite, MOR-1, (MOR-1/Clinoptilolite/Fe_3O_4)-10%CA.

Uv-vis characterization

UV–vis spectra of Bentonite, MOR-1 and (MOR-1/Bentonite)-HA were measured by the solid-state diffuse reflectance method and converted to absorption spectra with the Kubelka–Munk function. The intensity of all spectra has been normalized in order to be comparable. The spectrum of MOR-1 revealed two characteristic peaks at 240 and 370 nm attributed to a π-π^* transition of the ligand. In the bentonite spectrum, the peak at 240 nm corresponds to charge transfer from the oxygen atoms to the octahedral Fe(III), which may exist in the form of oxides in bentonite.[37] In the spectrum of the composite, two broad peaks at 240 nm and 370 nm are observed, which are almost identical to those of MOR-1. The absorption peak of bentonite cannot be distinguished in the spectrum of the composite as it coincides with that of MOR-1 at 240 nm. In the spectrum of Clinoptilolite, the peaks at 240 nm and 310 nm are attributed to the charge transfer from the oxygen atoms to tetrahedral and octahedral Al, respectively.[38–40] At 405 nm the absorption peak is due to various metal oxides such as iron oxides existing in clinoptilolite.[41,42] In the (MOR-1/Clinoptilolite)-HA composite, two peaks are observed at 240 nm and 370 nm (broad peak), which are due to the presence of both (MOR-1 and clinoptilolite) components.

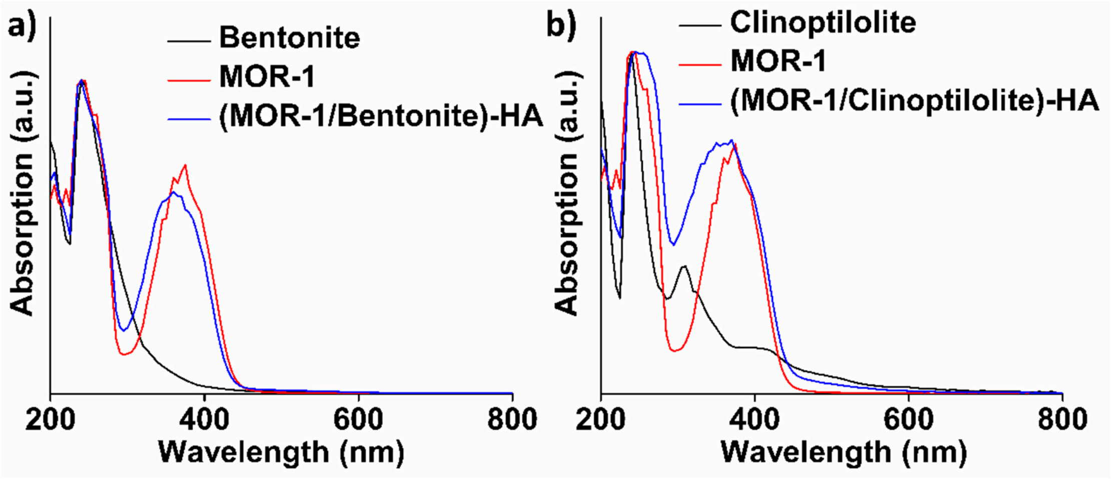

Figure S5. Solid state UV spectra of (**a**) bentonite, MOR-1, (MOR-1/Bentonite)-HA and (**b**) Clinoptilolite, MOR-1, (MOR-1/Clinoptilolite)-HA.

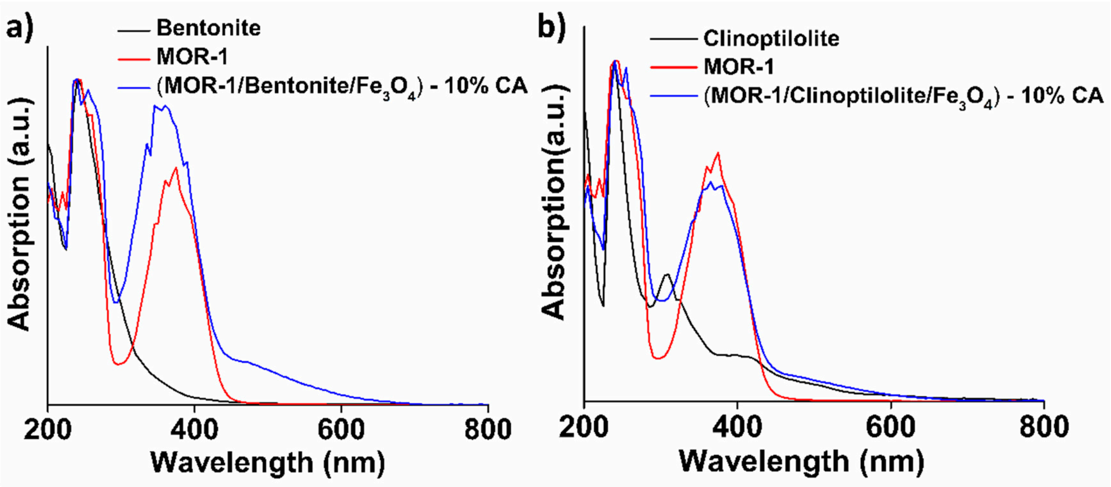

Figure S6. Solid state UV spectra of (**a**) bentonite, MOR-1, (MOR-1/Bentonite/Fe_3O_4)-10%CA and (**b**) clinoptilolite, MOR-1, (MOR-1/Clinoptilolite/Fe_3O_4)-10%CA.

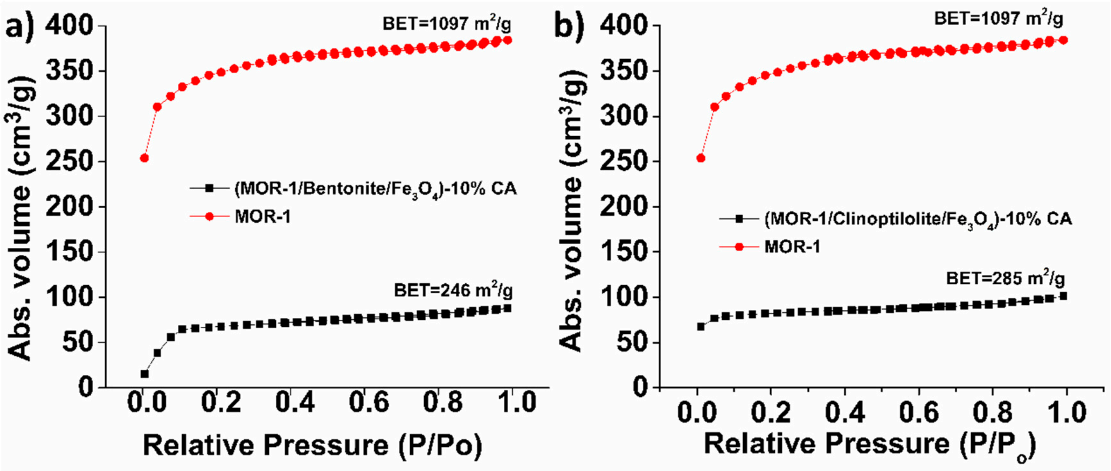

Figure S7. N_2 sorption isotherms (77 K) for MOR-1, (**a**) (MOR-1/Bentonite/Fe_3O_4)-10%CA and (**b**) (MOR-1/Clinoptilolite/Fe_3O_4)-10%CA.

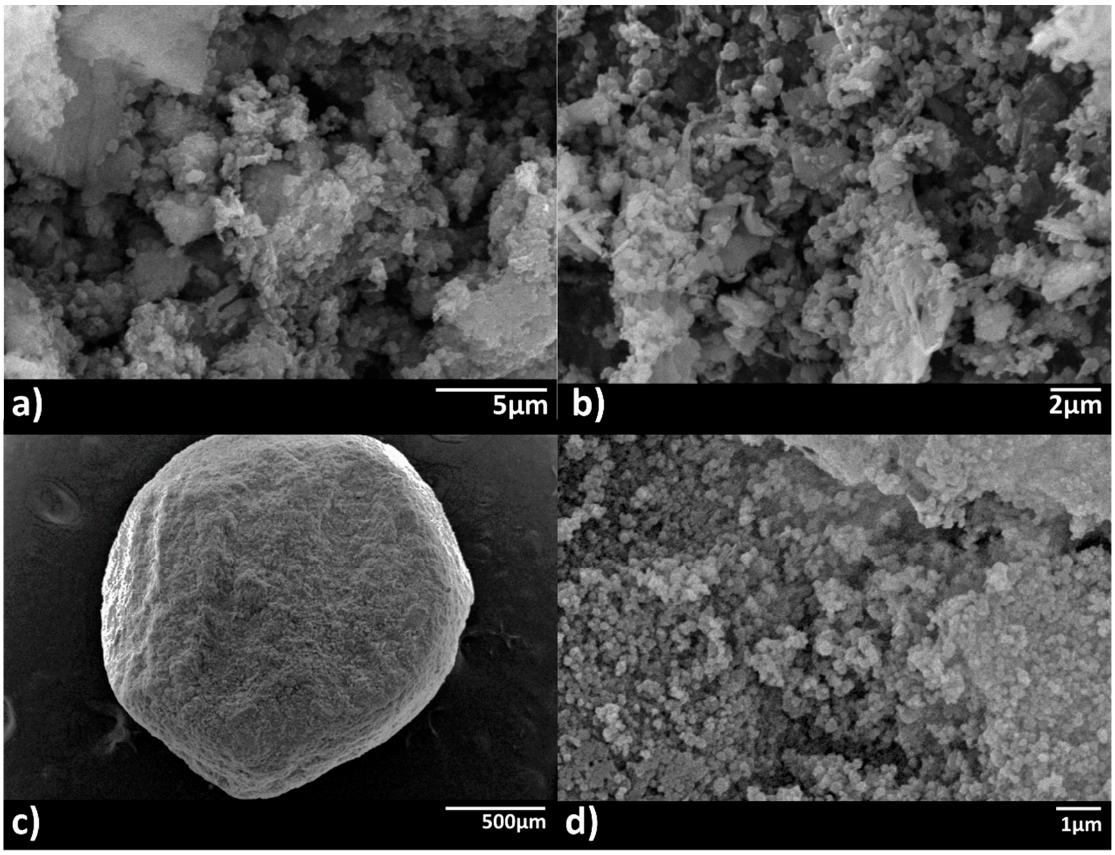

Figure S8. SEM images of (**a**) (MOR-1/Bentonite)-HA, (**b**) (MOR-1/Clinoptilolite)-HA, (**c**) (MOR-1/Bentonite/Fe_3O_4)-10%CA bead and (**d**) (MOR-1/Clinoptilolite/Fe_3O_4)-10%CA.

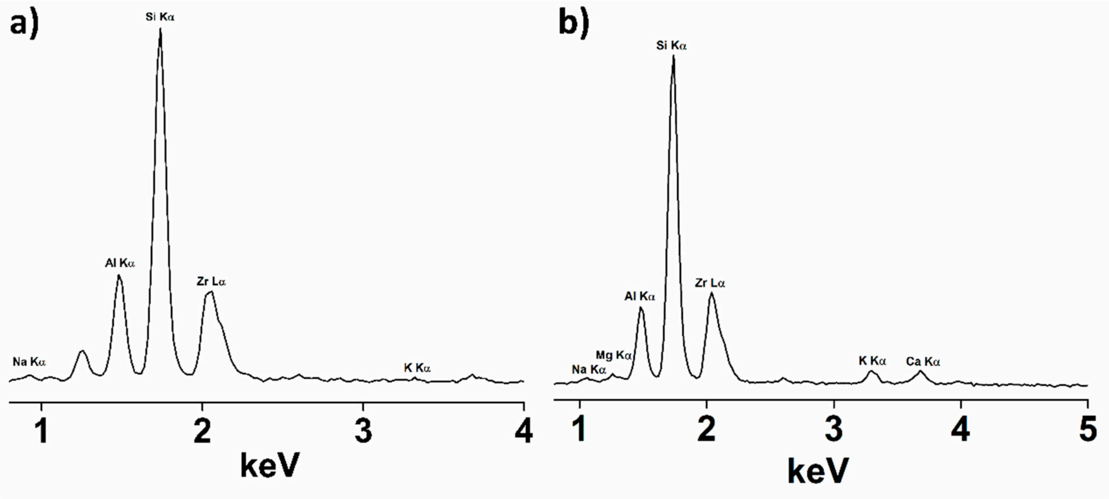

Figure S9. EDS spectra of (**a**) (MOR-1/Bentonite)-HA and (**b**) (MOR-1/Clinoptilolite)-HA.

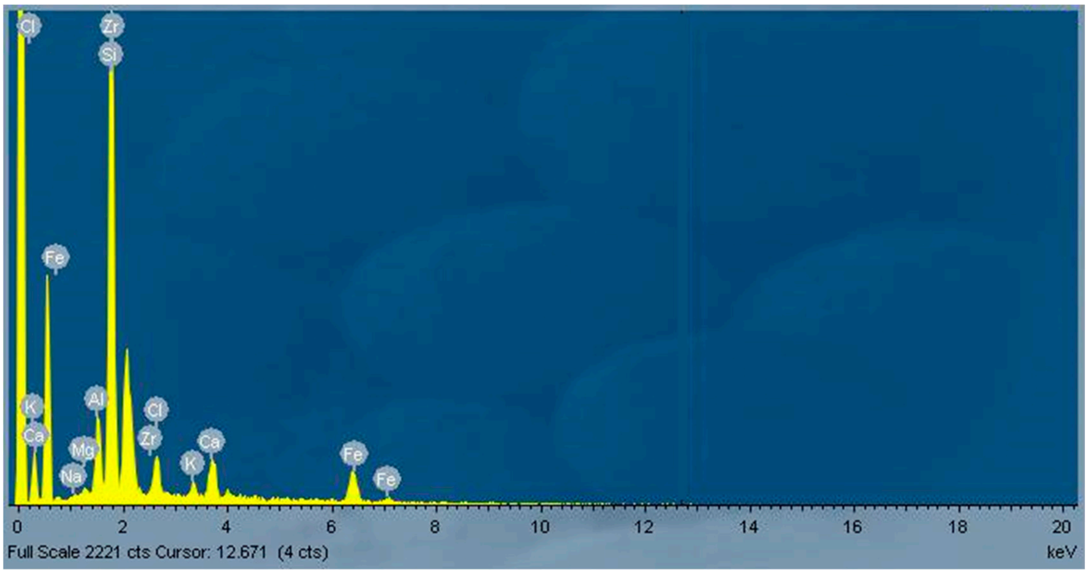

Figure S10. EDS spectrum of (MOR-1/Bentonite/Fe_3O_4)-10%CA.

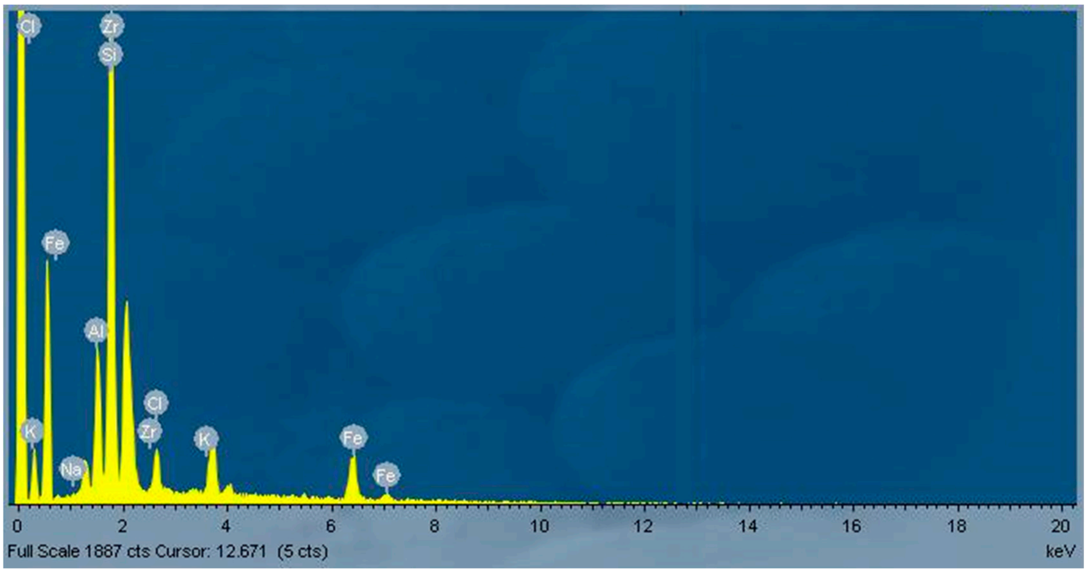

Figure S11. EDS spectrum of (MOR-1/Clinoptilolite/Fe_3O_4)-10%CA.

Batch ion-exchange studies

Lagergren's First-order equation and Ho-Mckay's pseudo-second-order equation were used to fit the kinetics data. The expressions of these equations are the following:

Lagergren's First-order equation:

$$q_t = q_e[1 - \exp(-K_L t)]$$

where q_e = the amount (mg g^{-1}) of ion sorbed in equilibrium, K_L = the Lagergren's or first-order rate constant.[9]

Ho and Mckay's pseudo-second-order equation:

$$q_t = \frac{k_2 q_e^2 t}{1 + k_2 q_e t}$$

where q_t = the amount (mg/g) of ion sorbed at different reaction times (t), q_e= the amount (mg/g) of ion sorbed in equilibrium, and where k_2 is the second-order rate constant [g/(mg·min)].[9]

Table S1. The parameters of Lagergren's first-order equation and Ho-Mckay's second-order equation, found after the fitting of kinetics data for the sorption of MB^+ and MO^- by the composite materials.

	Lagergren's First-Order Equation				*Ho-Mckay's Second-Order Equation*			
	q_e mg g^{-1}	K_L g (mg min)$^{-1}$	R^2	RSS	q_e mg g^{-1}	K_2 g (mg min)$^{-1}$	R^2	RSS
MOR-1-Bentonite-HA@MB⁺	22.71 ± 0.05	3.45 ± 0.21	0.70	0.17	22.88 ± 0.02	1.09 ± 0.08	0.95	0.02
MOR-1-Bentonite-HA@MO⁻	21.84 ± 0.07	3.09 ± 0.21	0.71	0.30	22.08 ± 0.03	0.78 ± 0.06	0.96	0.04
MOR-1-Clinoptinolite-HA@MB⁺	21.90 ± 0.02	4.66 ± 0.29	0.59	0.002	21.95 ± 0.01	3.62 ± 0.22	0.97	0.0001
MOR-1-Clinoptinolite-HA@MO⁻	21.81 ± 0.04	4.23 ± 0.35	0.48	0.08	21.89 ± 0.02	2.28 ± 0.25	0.92	0.01

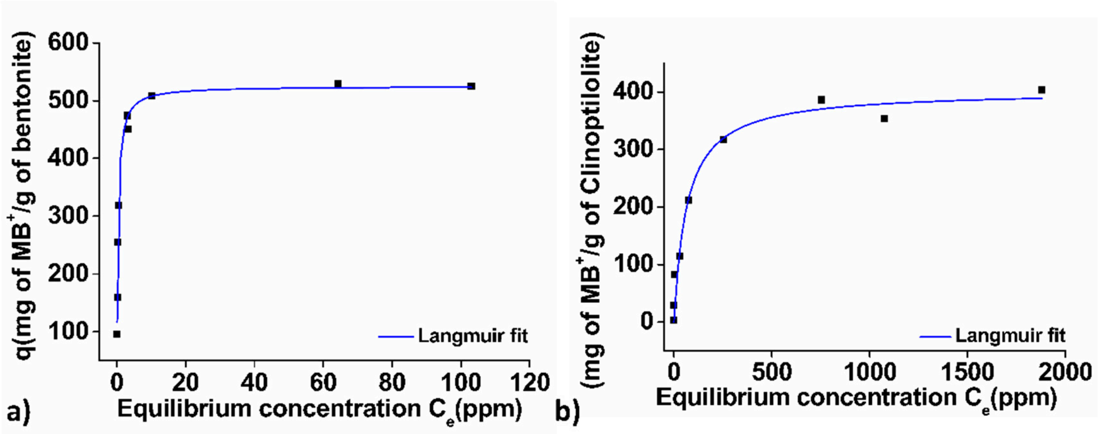

Figure S12. MB^+ isotherm sorption data for (**a**) bentonite and (**b**) clinoptilolite.

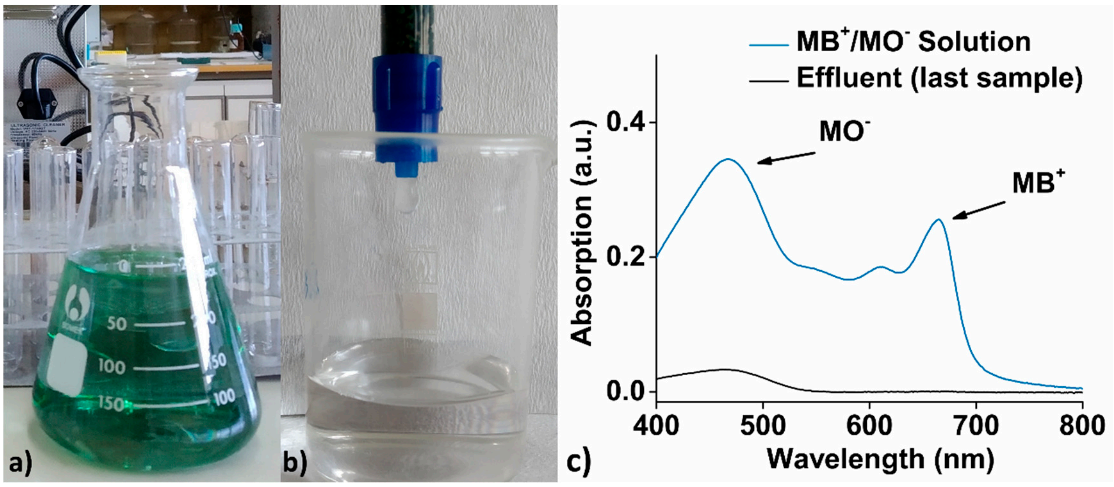

Figure S13. (**a**) MB^+/MO^- mixture solution of initial concentration of 3.7 ppm MB^+ and 5.3 ppm MO^+. (**b**) The effluent of the column is colorless. (**c**) UV spectra of the MB^+/MO^- mixture solution before (blue line) and after passing through the column (black line).

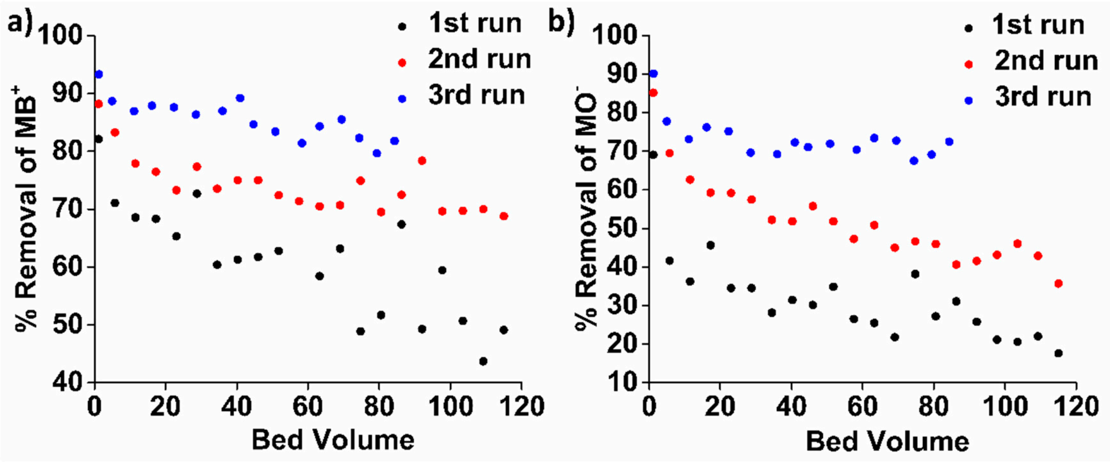

Figure S14. Column sorption data for the removal of (**a**) MB^+ and (**b**) MO^- by (MOR-1/Bentonite/Fe_3O_4)-10%CA beads.

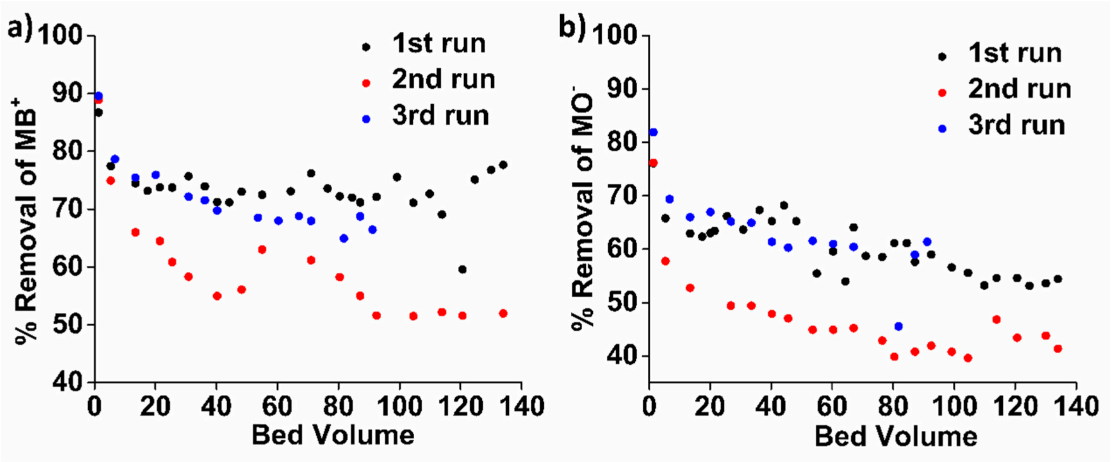

Figure S15. Column sorption data for the removal of (**a**) MB^+ and (**b**) MO^- by (MOR-1/Clinoptilolite/Fe_3O_4)-10%CA beads.

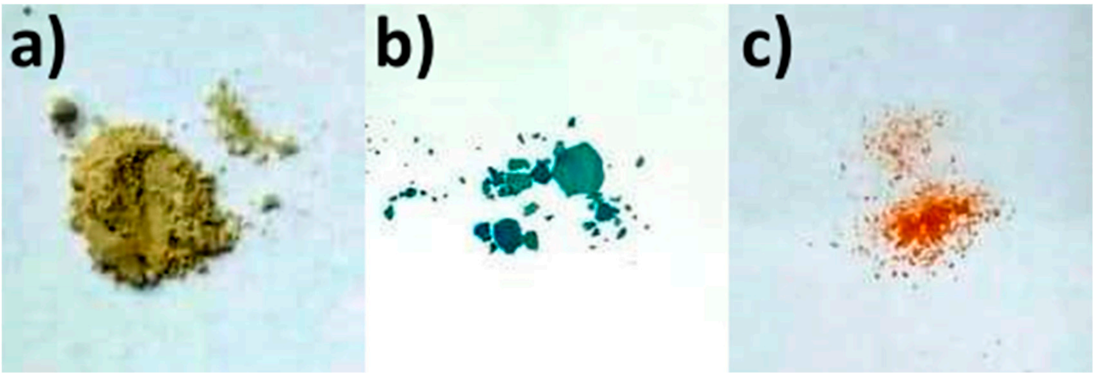

Figure S16. Optical images of (**a**) (MOR-1/Bentonite)-HA, (**b**) (MOR-1/Bentonite)-HA@MB^+ and (**c**) (MOR-1/Bentonite)-HA@MO^-.

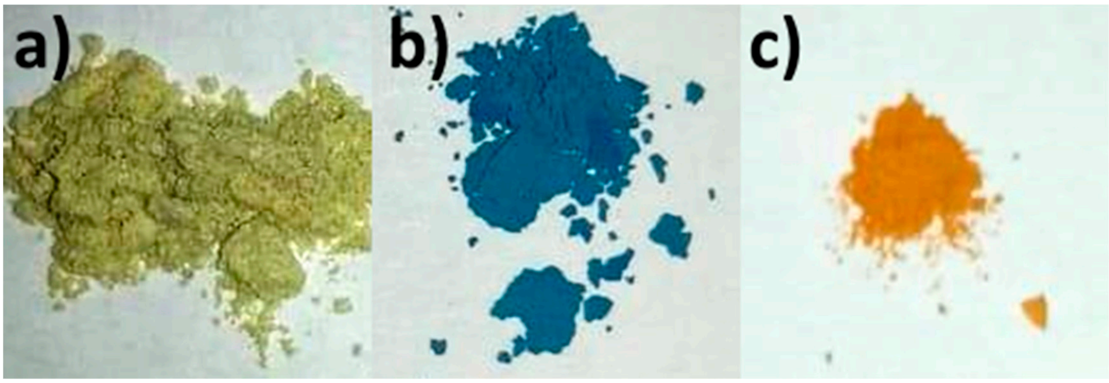

Figure S17. Optical images of (**a**) (MOR-1/Clinoptilolite)-HA, (**b**) (MOR-1/ Clinoptilolite)-HA@MB⁺ and (**c**) (MOR-1/ Clinoptilolite)-HA@MO⁻.

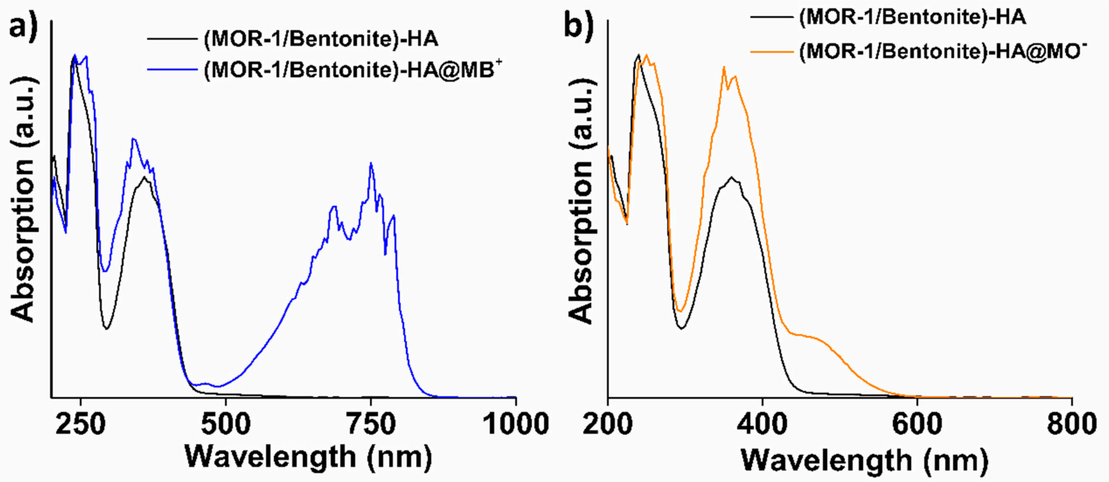

Figure S18. Solid state UV spectra of (MOR-1/Bentonite)-HA in comparison with (**a**) (MOR-1/Bentonite)-HA@MB⁺ and (**b**) (MOR-1/Bentonite)-HA@MO⁻.

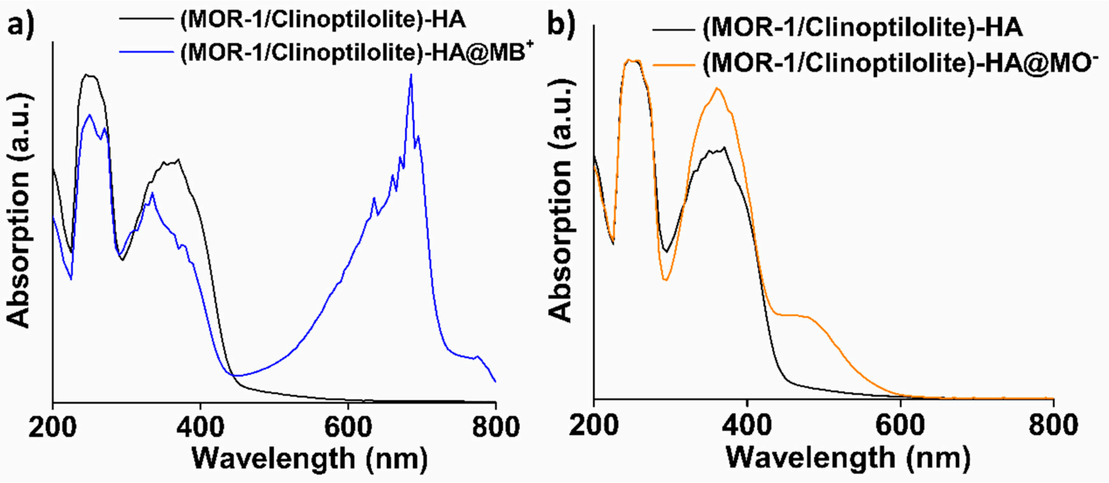

Figure S19. Solid state UV spectra of (MOR-1/Clinoptilolite)-HA in comparison with (**a**) (MOR-1/ Clinoptilolite)-HA@MB⁺ and (**b**) (MOR-1/ Clinoptilolite)-HA@MO⁻.

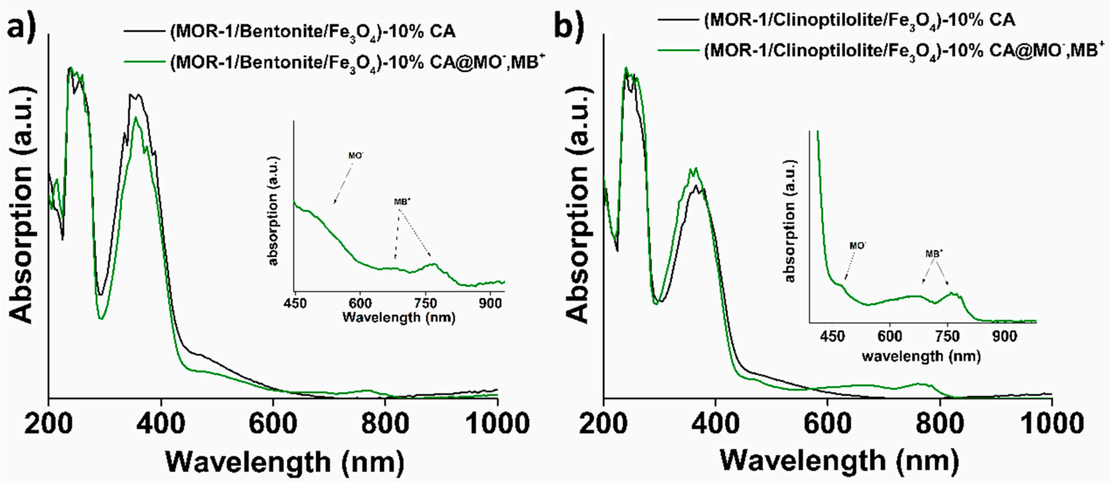

Figure S20. Solid state UV spectra of (**a**) (MOR-1/Bentonite/Fe_3O_4)-10%CA and (**b**) (MOR-1/Clinoptilolite/Fe_3O_4)-10%CA, before and after the sorption of the MB^+ and MO^-.

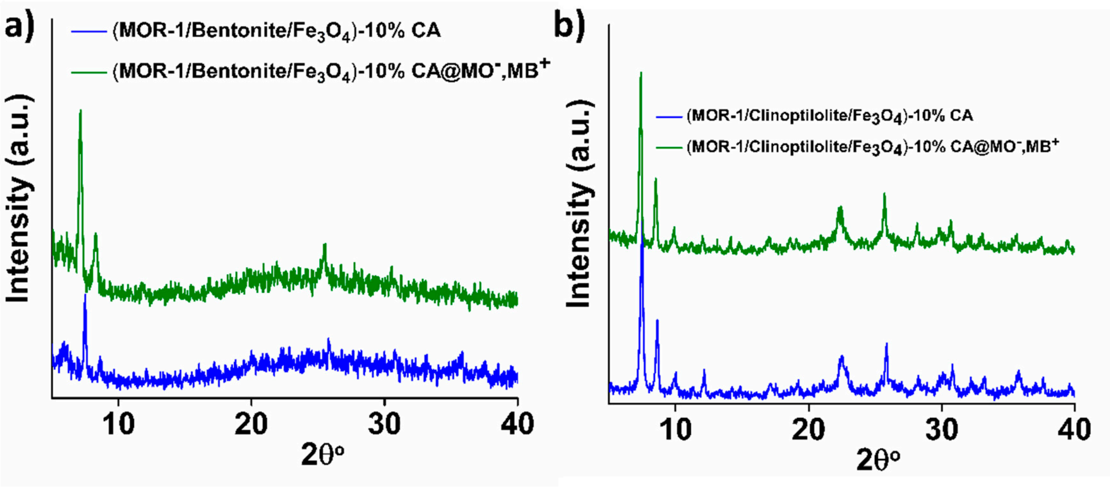

Figure S21. PXRD patterns of (**a**) (MOR-1/Bentonite/Fe_3O_4)-10%CA and (**b**) (MOR-1/Clinoptilolite/Fe_3O_4)-10%CA, before and after the sorption of the MB^+ and MO^-.

References

1. Petrie, B.; Barden, R.; Kasprzyk-Hordern, B. A review on emerging contaminants in wastewaters and the environment: Current knowledge, understudied areas and recommendations for future monitoring. *Water Res.* **2015**, *72*, 3–27.
2. Bhatia, D.; Sharma, N.R.; Singh, J.; Kanwar, R.S. Biological methods for textile dye removal from wastewater: A review. *Crit. Rev. Environ. Sci. Technol.* **2017**, *47*, 1836–1876.
3. Jamee, R.; Siddique, R. Biodegradation of synthetic dyes of textile effluent by microorganisms: An environmentally and economically sustainable approach. *Eur. J. Microbiol. Immunol.* **2019**, *9*, 114–118.
4. Yang, C.; Li, L.; Shi, J.; Long, C.; Li, A. Advanced treatment of textile dyeing secondary effluent using magnetic anion exchange resin and its effect on organic fouling in subsequent RO membrane. *J. Hazard. Mater.* **2015**, *284*, 50–57.
5. Rauf, M.A.; Salman Ashraf, S. Survey of recent trends in biochemically assisted degradation of dyes. *Chem. Eng. J.* **2012**, *209*, 520–530.
6. Golka, K.; Kopps, S.; Myslak, Z.W. Carcinogenicity of azo colorants: Influence of solubility and bioavailability. *Toxicol. Lett.* **2004**, *151*, 203–210.
7. Haque, E.; Lo, V.; Minett, A.I.; Harris, A.T.; Church, T.L. Dichotomous adsorption behaviour of dyes on an amino-functionalised metal-organic framework, amino-MIL-101(Al). *J. Mater. Chem. A* **2014**, *2*, 193–203.
8. Robinson, T.; McMullan, G.; Marchant, R.; Nigam, P. Remediation of dyes in textile effluent: A critical review on current treatment technologies with a proposed alternative. *Bioresour. Technol.* **2001**, *77*, 247–255.
9. Mezohegyi, G.; van der Zee, F.P.; Font, J.; Fortuny, A.; Fabregat, A. Towards advanced aqueous dye removal processes: A short review on the versatile role of activated carbon. *J. Environ. Manage.* **2012**, *102*, 148–164.
10. Sarro, M.; Gule, N.P.; Laurenti, E.; Gamberini, R.; Paganini, M.C.; Mallon, P.E.; Calza, P. ZnO-based materials and enzymes hybrid systems as highly efficient catalysts for recalcitrant pollutants abatement. *Chem. Eng. J.* **2018**, *334*, 2530–2538.

11. Tang, L.; Yu, J.; Pang, Y.; Zeng, G.; Deng, Y.; Wang, J.; Ren, X.; Ye, S.; Peng, B.; Feng, H. Sustainable efficient adsorbent: Alkali-acid modified magnetic biochar derived from sewage sludge for aqueous organic contaminant removal. *Chem. Eng. J.* **2018**, *336*, 160–169.
12. Pournara, A.D.; Rapti, S.; Skliri, E.; Armatas, G.S.; Tsipis, A.C.; Manos, M.J. Highly Efficient Sorption of Methyl Orange by a Metal–Organic Resin–Alginic Acid Composite. *Chempluschem* **2017**, *82*, 1188–1196.
13. Li, J.; Wang, X.; Zhao, G.; Chen, C.; Chai, Z.; Alsaedi, A.; Hayat, T.; Wang, X. Metal-organic framework-based materials: Superior adsorbents for the capture of toxic and radioactive metal ions. *Chem. Soc. Rev.* **2018**, *47*, 2322–2356.
14. Kumar, P.; Pournara, A.; Kim, K.H.; Bansal, V.; Rapti, S.; Manos, M.J. Metal-organic frameworks: Challenges and opportunities for ion-exchange/sorption applications. *Prog. Mater. Sci.* **2017**, *86*, 25–74.
15. Kang, K.; Liu, S.; Zhang, M.; Li, L.; Liu, C.; Lei, L.; Dai, X.; Xu, C.; Xiao, C. Fast Room-Temperature Synthesis of an Extremely Alkaline-Resistant Cationic Metal–Organic Framework for Sequestering TcO_4^- with Exceptional Selectivity. *Adv. Funct. Mater.* **2022**, *32*, 2208148.
16. Kang, K.; Shen, N.; Wang, Y.; Li, L.; Zhang, M.; Zhang, X.; Lei, L.; Miao, X.; Wang, S.; Xiao, C. Efficient sequestration of radioactive $^{99}TcO_4^-$ by a rare 3-fold interlocking cationic metal-organic framework: A combined batch experiments, pair distribution function, and crystallographic investigation. *Chem. Eng. J.* **2022**, *427*, 130942.
17. Kausar, A.; Iqbal, M.; Javed, A.; Aftab, K.; Nazli, Z.I.H.; Bhatti, H.N.; Nouren, S. Dyes adsorption using clay and modified clay: A review. *J. Mol. Liq.* **2018**, *256*, 395–407.
18. Wang, S.; Peng, Y. Natural zeolites as effective adsorbents in water and wastewater treatment. *Chem. Eng. J.* **2010**, *156*, 11–24.
19. Zagorodni, A.A. *Ion Exchange Materials: Properties and Applications*; Elsevier: Amsterdam, The Netherlands, 2007; p. 280.
20. Rapti, S.; Pournara, A.; Sarma, D.; Papadas, I.T.; Armatas, G.S.; Tsipis, A.C.; Lazarides, T.; Kanatzidis, M.G.; Manos, M.J. Selective capture of hexavalent chromium from an anion-exchange column of metal organic resin-alginic acid composite. *Chem. Sci.* **2016**, *7*, 2427–2436.
21. Rapti, S.; Pournara, A.; Sarma, D.; Papadas, I.T.; Armatas, G.S.; Hassan, Y.S.; Alkordi, M.H.; Kanatzidis, M.G.; Manos, M.J. Rapid, green and inexpensive synthesis of high quality UiO-66 amino-functionalized materials with exceptional capability for removal of hexavalent chromium from industrial waste. *Inorg. Chem. Front.* **2016**, *3*, 635–644.
22. Rapti, S.; Diamantis, S.A.; Dafnomili, A.; Pournara, A.; Skliri, E.; Armatas, G.S.; Tsipis, A.C.; Spanopoulos, I.; Malliakas, C.D.; Kanatzidis, M.G.; et al. Exceptional TcO_4^- sorption capacity and highly efficient ReO_4^- luminescence sensing by Zr^{4+} MOFs. *J. Mater. Chem. A* **2018**, *6*, 20813–20821.
23. Pournara, A.D.; Rapti, S.; Valmas, A.; Margiolaki, I.; Andreou, E.; Armatas, G.S.; Tsipis, A.C.; Plakatouras, J.C.; Giokas, D.L.; Manos, M.J. Alkylamino-terephthalate ligands stabilize 8-connected Zr^{4+} MOFs with highly efficient sorption for toxic Se species. *J. Mater. Chem. A* **2021**, *9*, 3379–3387.
24. Pournara, A.D.; Evangelou, D.A.; Roukounaki, C.; Andreou, E.K.; Armatas, G.S.; Lazarides, T.; Manos, M.J. Highly efficient sorption and luminescence sensing of oxoanionic species by 8-connected alkyl-amino functionalized Zr^{4+} MOFs. *Dalt. Trans.* **2022**, *51*, 17301–17309.
25. Pournara, A.D.; Rizogianni, S.; Evangelou, D.A.; Andreou, E.K.; Armatas, G.S.; Manos, M.J. Zr^{4+} -terephthalate MOFs with 6-connected structures, highly efficient As (III/V) sorption and superhydrophobic properties. *Chem. Commun.* **2022**, *58*, 8862–8865.
26. Diamantis, S.A.; Pournara, A.D.; Koutsouroubi, E.D.; Moularas, C.; Deligiannakis, Y.; Armatas, G.S.; Hatzidimitriou, A.G.; Manos, M.J.; Lazarides, T. Detection and Sorption of Heavy Metal Ions in Aqueous Media by a Fluorescent Zr(IV) Metal–Organic Framework Functionalized with 2-Picolylamine Receptor Groups. *Inorg. Chem.* **2022**, *61*, 7847–7858.
27. Kubilay, Ş.; Gürkan, R.; Savran, A.; Şahan, T. Removal of Cu(II), Zn(II) and Co(II) ions from aqueous solutions by adsorption onto natural bentonite. *Adsorption* **2007**, *13*, 41–51.
28. Tahir, S.S.; Rauf, N. Removal of a cationic dye from aqueous solutions by adsorption onto bentonite clay. *Chemosphere* **2006**, *63*, 1842–1848.
29. Qiu, M.; Qian, C.; Xu, J.; Wu, J.; Wang, G. Studies on the adsorption of dyes into clinoptilolite. *Desalination* **2009**, *243*, 286–292.
30. Smičiklas, I.; Dimović, S.; Plećaš, I. Removal of Cs^{1+}, Sr^{2+} and Co^{2+} from aqueous solutions by adsorption on natural clinoptilolite. *Appl. Clay Sci.* **2007**, *35*, 139–144.
31. Cadar, O.; Senila, M.; Hoaghia, M.A.; Scurtu, D.; Miu, I.; Levei, E.A. Effects of thermal treatment on natural clinoptilolite-rich zeolite behavior in simulated biological fluids. *Molecules* **2020**, *25*, 2570–2582.
32. Eren, E.; Afsin, B. An investigation of Cu(II) adsorption by raw and acid-activated bentonite: A combined potentiometric, thermodynamic, XRD, IR, DTA study. *J. Hazard. Mater.* **2008**, *151*, 682–691.
33. Sahoo, T.R.; Prelot, B. *Nanomaterials for the Detection and Removal of Wastewater Pollutants*; Elsevier: Amsterdam, The Netherlands,2020.
34. Manos, M.J.; Kanatzidis, M.G. Sequestration of heavy metals from water with layered metal sulfides. *Chem.—A Eur. J.* **2009**, *15*, 4779–4784.
35. Wang, J.; Guo, X. Adsorption isotherm models: Classification, physical meaning, application and solving method, *Chemosphere* **2020**, *258*, 127279.
36. Hadi, M.; Samarghandi, M.R.; McKay, G. Simplified fixed bed design models for the adsorption of acid dyes on novel pine cone derived activated carbon. *Water. Air. Soil Pollut.* **2011**, *218*, 197–212.
37. Karickhoff, S.W.; Bailey, G.W. Optical absorption spectra of clay minerals. *Clays Clay Miner.* **1973**, *21*, 51–57.

38. Garbowski, E.D.; Mirodatos, C. Investigation of structural charge transfer in zeolites by ultraviolet spectroscopy. *J. Phys. Chem.* **1982**, *86*, 97–102.
39. Garcia-Basabe, Y.; Rodriguez-Iznaga, I.; De Menorval, L.C.; Llewellyn, P.; Maurin, G.; Lewis, D.W.; Binions, R.; Autie, M.; Ruiz-Salvador, A.R. Step-wise dealumination of natural clinoptilolite: Structural and physicochemical characterization. *Microporous Mesoporous Mater*. **2010**, *135*, 187–196.
40. 6Zanjanchi, M.A.; Razavi, A. Identification and estimation of extra-framework aluminium in acidic mazzite by diffuse reflectance spectroscopy. *Spectrochim. Acta - Part A Mol. Biomol. Spectrosc*. **2001**, *57*, 119–127.
41. Bordiga, S.; Buzzoni, R.; Geobaldo, F.; Lamberti, C.; Giamello, E.; Zecchina, A.; Leofanti, G.; Petrini, G.; Tozzola, G.; Vlaic, G. Structure and reactivity of framework and extraframework iron in Fe-silicalite as investigated by spectroscopic and physicochemical methods. *J. Catal.* **1996**, *158*, 486–501.
42. Pérez-Ramírez, J.; Kumar, M.S.; Brückner, A. Reduction of N2O with CO over FeMFI zeolites: Influence of the preparation method on the iron species and catalytic behavior. *J. Catal*. **2004**, *223*, 13–27.
43. Benhammou, A.; Yaacoubi, A.; Nibou, L.; Tanouti, B. Adsorption of metal ions onto Moroccan stevensite: Kinetic and isotherm studies. *J. Colloid Interface Sci.* **2005**, *282*, 320–326.
